# lncRNA Helf promotes hepatic inflammation and fibrosis by interacting with PTBP1 to facilitate PIK3R5 mRNA stabilization

**DOI:** 10.1186/s11658-023-00492-3

**Published:** 2023-10-07

**Authors:** Xiaohui Han, Beichen Guo, Sicong Zhao, Yehua Li, Jing Zhu, Yifan He, Jiajun Wang, Qingbin Yao, Shuai Shao, Lina Zheng, Zhemin Shi, Tao Han, Wei Hong, Kun Zhang

**Affiliations:** 1 Department of Histology and Embryology, School of Basic Medical Sciences, Tianjin Medical University, 22 Qixiangtai Road, Tianjin, 300070 China; 2 Department of Hepatology and Gastroenterology, Tianjin Union Medical Center Affiliated to Nankai University, Tianjin, China

**Keywords:** lncRNA Helf, Hepatic fibrosis, Inflammation, PTBP1, PI3K/AKT pathway

## Abstract

**Background:**

Hepatic fibrosis is a common consequence of chronic liver diseases without approved antifibrotic therapies. Long noncoding RNAs (lncRNAs) play an important role in various pathophysiological processes. However, the functions of certain lncRNAs involved in mediating the antifibrotic role remain largely unclear.

**Methods:**

The RNA level of lnc-High Expressed in Liver Fibrosis (Helf) was detected in both mouse and human fibrotic livers. Furthermore, lnc-Helf-silenced mice were treated with carbon tetrachloride (CCl_4_) or bile duct ligation (BDL) to investigate the function of lnc-Helf in liver fibrosis.

**Results:**

We found that lnc-Helf has significantly higher expression in human and mouse fibrotic livers as well as M1 polarized hepatic macrophages (HMs) and activated hepatic stellate cells (HSCs). In vivo studies showed that silencing lnc-Helf by AAV8 vector alleviates CCl_4_- and BDL-induced hepatic inflammation and fibrosis. Furthermore, in vitro experiments revealed that lnc-Helf promotes HSCs activation and proliferation, as well as HMs M1 polarization and proliferation in the absence or presence of cytokine stimulation. Mechanistically, our data illustrated that lnc-Helf interacts with RNA binding protein PTBP1 to promote its interaction with PIK3R5 mRNA, resulting in increased stability and activating the AKT pathway, thus promoting HSCs and HMs activation and proliferation, which augments hepatic inflammation and fibrosis.

**Conclusion:**

Our results unveil a lnc-Helf/PTBP1/PIK3R5/AKT feedforward, amplifying signaling that exacerbates the process of hepatic inflammation and fibrosis, thus providing a possible therapeutic strategy for hepatic fibrosis.

**Supplementary Information:**

The online version contains supplementary material available at 10.1186/s11658-023-00492-3.

## Background

Hepatic fibrosis is a common consequence of chronic liver diseases caused by various etiologies, such as chronic viral hepatitis B and C, cholestasis, or alcohol abuse [1, 2]. If not resolved, the fibrotic process leads to cirrhosis and liver failure, which causes more than 1 million deaths annually worldwide [1, 2]. Liver transplantation, the definitive and sole treatment option for end-stage hepatic fibrosis, is limited by the shortage of available donors [1, 2]. Therefore, a more detailed understanding of the pathophysiological mechanism of hepatic fibrosis is needed to develop antifibrotic therapeutic strategies.

Fibrosis is characterized by the excessive deposition of extracellular matrix (ECM) components [1, 2]. Activated hepatic stellate cells (HSCs), the primary ECM-producing cells in the liver, respond to and secrete multifarious profibrotic cytokines, such as transforming growth factor beta (TGF-β) and platelet-derived growth factor (PDGF), all of which are the powerful cytokines resulting in hepatic fibrosis [3, 4]. Besides HSCs, accumulating evidences also demonstrate that hepatic macrophages (HMs), which consist of liver-resident Kupffer cells and bone marrow monocyte-derived macrophages (BMMs) that are recruited from the circulation upon liver injury, are crucial to the progression of liver fibrosis [5, 6]. It is known that HMs release various factors, including TGF-β, tumor necrosis factor alpha (TNF-α), and interleukin 1 beta (IL-1β), and subsequently cause direct damage to hepatocyte (HC), facilitating inflammatory cell infiltration and activation of HSCs [5]. Thus, a more in-depth understanding of the molecular mechanism involved in HM-orchestrated hepatic inflammation and subsequent interplay with HSCs is vital to develop effective therapeutic strategies.

The enormous transcripts transcribed from the mammalian genome are non-coding RNAs (ncRNAs) [7]. Although some of these ncRNAs, including snoRNA, miRNA, rRNA, and tRNA, have been intensely studied, only a minority of long non-coding RNAs (lncRNAs) have confident annotations and very few have mechanistic information [7, 8]. Current researches indicate that lncRNAs participate in various biological processes and human diseases through regulating the expression of targeted genes at the transcriptional, epigenetic, and post-transcriptional levels [9–13]. Recently, we and others have demonstrated that some of lncRNAs play a major role in HSCs activation, hepatocyte apoptosis and hepatic fibrosis [14–18]. For instance, we have identified that the liver-enriched lnc-Lfar1 without human orthologs promotes HSCs activation and HC apoptosis through activating the TGF-β and Notch pathways [14]. Further investigation demonstrated that silencing lnc-Lfar1 alleviates carbon tetrachloride (CCl_4_) or bile duct ligation (BDL)-induced proinflammatory M1 macrophage polarization and pyroptosis [15]. Moreover, we found that nuclear-retained lncRNA SCARNA10, which has human and mouse homology, is upregulated in serum of patients with liver fibrosis and functions as a positive regulator of liver fibrosis by suppressing polycomb repressive complex 2 (PRC2) and interacts with the promoters of ECM genes [16]. In addition, we also identified that hepatocyte-specifically expressed lnc-Hser inhibits HC apoptosis through the C5AR1-YAP signaling and suppresses HC epithelial–mesenchymal transition via the Notch pathway [17]. Despite this knowledge, the functions of certain lncRNAs involved in mediating the antifibrotic role have yet to be clarified.

In this study, we identified a novel lnc-High Expressed in Liver Fibrosis (Helf) that is increased in mouse and human fibrotic livers and systemically investigated its regulatory and functional role in hepatic inflammation and fibrosis.

## Materials and methods

### Clinical specimens

Study population analysis was conducted as previously reported [17]. Briefly, 6 normal livers and 28 human fibrotic livers were obtained from Tianjin Third Central Hospital (Tianjin, China).

Three pathologists blinded to the study protocol scored liver fibrosis according to the meta-analysis of histological data in viral hepatitis (METAVIR) fibrosis staging system. The clinical characteristics were summarized in Additional file 1: Table S1. The research methods were accorded with the standards in the Helsinki Declaration. Written informed consents were acquired from each patient and the study has been approved by the local Ethical Committee of Tianjin Third Central Hospital (Tianjin, China).

### Animal in vivo study

All animal work was conducted according to the guidelines approved by the Animal Care and Use Committee of Tianjin Medical University. Eight-week-old Balb/c male mice were purchased from Beijing HFK bioscience (Beijing, China). For hepatic fibrosis model induced by CCl_4_, 60 mice were randomly separated into six groups: AAV8-negative control (NC), NC + CCl_4_, AAV-lnc-Helf-shRNA-1#, AAV-lnc-Helf-shRNA-1# + CCl_4_, AAV-lnc-Helf-shRNA-2#, and AAV-lnc-Helf-shRNA-2# + CCl_4_ (*n* = 10). The adeno-associated virus was injected 2 weeks after the first injection of CCl_4_ via the tail vein (2 × 10^11^ pfu/mouse). NC + CCl_4_ group, lnc-Helf-sh1# + CCl_4_, and lnc-Helf-sh2# + CCl_4_ group mice were treated with CCl_4_ (0.2 ml/kg) twice per week for additional 6 weeks after the virus was injected. AAV8-NC, AAV-lnc-Helf-shRNA-1# and AAV-lnc-Helf-shRNA-2# group mice were injected with an equivalent amount of olive oil. For hepatic fibrosis model induced by BDL, 90 Balb/c mice were randomly separated into six groups: AAV8-NC, NC + BDL, lnc-Helf-sh1#, lnc-Helf-sh1# + BDL, lnc-Helf-sh2#, and lnc-Helf-sh2# + BDL (*n* = 15). Mice were injected with AAV8-NC or AAV8-lnc-Helf-shRNAs virus (2 × 10^11^ pfu/mouse) via tail vein 2 days before sham operation or bile duct ligate operation. A total of 21 days after the operation, serum and liver specimens were collected for further analyses.

### RNA-Seq and computational analysis

RNA harvested from liver tissues of mice treated with NC + CCl_4_, AAV-lnc-Helf-shRNA-1# + CCl_4_, and AAV-lnc-Helf-shRNA-2# + CCl_4_ (*n* = 3) was qualified by Agilent 2100 bioanalyzer (Thermo, USA) and subsequently screened on the BGIseq500 platform (BGI-Shenzhen, China). The threshold was fold change > 2 and *p*_adj_ < 0.05. The sequencing data have been deposited in NCBI GEO database: GSE182485 (https://www.ncbi.nlm.nih.gov/geo/query/acc.cgi?acc=GSE182485).

### Primary HSCs and HMs isolation

Mouse primary HSCs were isolated from 40-week-old male Balb/c mice by in situ ethylene glycol tetra-acetic acid (EGTA)/Hank’s balanced salt solution (HBSS) solution, pronase, collagenase perfusion, further digestion, and Histodenz gradient centrifugation as previously reported [14, 19]. Moreover, the purity of HSCs was detected by alpha-smooth muscle actin (α-SMA) staining and perinuclear lipid droplets. Mouse primary HMs were isolated from the 10-week-old male Balb/c mice by in situ EGTA/HBSS solution, collagenase perfusion, further digestion, and Histodenz gradient centrifugation as previously reported [15]. Selective adhesion or magnetic-activated cell sorting (MACS)-based positive selection using a F4/80 antibody (eBioscience) was used to purify HMs. Cell viability was measured by Trypan blue exclusion assay. Primary HSCs and HMs were cultured in high-glucose Dulbecco’s modified Eagle medium (DMEM) containing 10% fetal bovine serum (FBS) and 1% penicillin/streptomycin.

### Biotinylated RNA pull-down assay

For biotinylated RNA pull-down assay, lnc-Helf was transcribed in vitro with T7 RNA polymerase (Thermo, #k0441). Then, T4 RNA ligase was used to attach a single biotinylated nucleotide to the 3′ terminus of an RNA strand at 16 °C for 16 h (Thermo, 20163). The labeled RNA was bound to the Streptavidin Magnetic Beads at 25 °C incubating for 30 min. The mixture was washed with 20 mM Tris (pH 7.5) two times. Liver single cell suspensions of mice were prepared and total of 2 × 10^7^ cells were lysed. RNA-bound beads were mixed to the lysates and incubated for 1 h at 4 °C. Followed by washing with wash buffer, we added elution buffer to the beads, mixed and incubated at 37 °C with agitation for 30 min. Supernatant was removed for downstream analysis (Thermo, 20164).

### RNA immunoprecipitation (RIP)

RIP was performed using the Magna RIP RNA-Binding Protein Immunoprecipitation Kit (Millipore, No.17-700, Bedford, MA, USA) according to the manufacturer’s instructions. Briefly, mouse liver single cell suspensions and RAW 264.7 cells were lysed by RIP lysis buffer and stored at −80 °C. A total of 5 µg of the antibody (anti-PTBP1 and control IgG) was added to magnetic beads and incubated with rotation for 30 min. Then, the RIP lysate was centrifuged at 14,000 rpm for 10 min at 4 °C. The beads were added to the lysates and RIP immunoprecipitation buffer and incubated at 4 °C for overnight. Next, RIP buffer was briefly centrifuged to remove the supernatant. The purified RNAs were used for cDNA synthesis and evaluated by quantitative reverse transcription polymerase chain reaction (qRT–PCR).

### Statistical analysis

Data were expressed as mean ± standard error of the mean (SEM) with at least three independent experiments. All statistical analyses were conducted using SPSS 23.0 (IBM, Armonk, NY, USA). Statistical analysis was conducted using either one-way analysis of variance (more than two groups) or Student’s *t*-test (two group comparison), and *p* < 0.05 indicated a significant difference.

Details on other materials and methods are provided in the Additional file 1.

## Results

### lnc-Helf is increased in mouse and human hepatic fibrosis

**Fig. 1:**
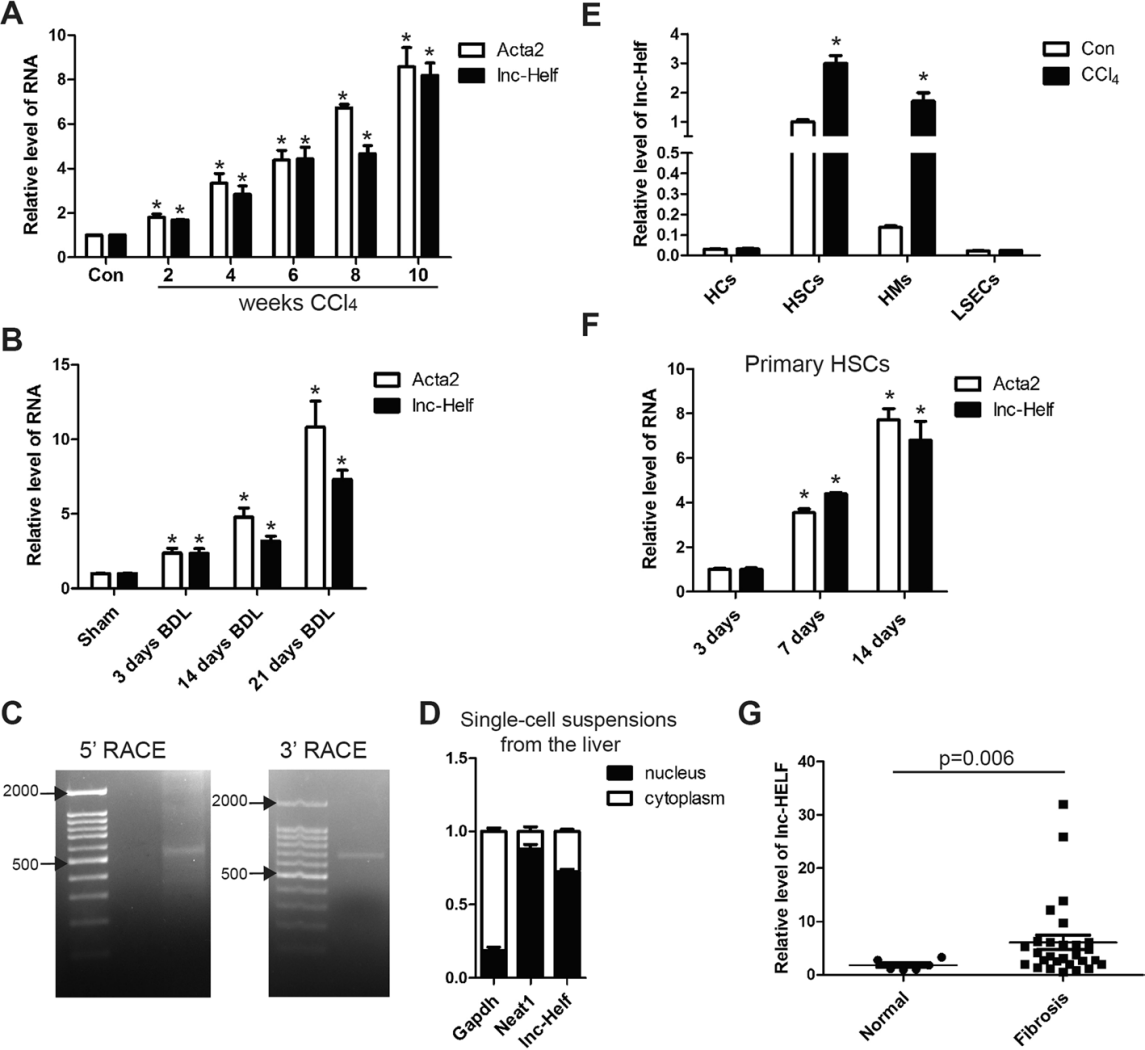
lnc-Helf is increased in mouse and human hepatic fibrosis. **A**, **B** The expression of *lnc-Helf* and *Acta2 (α-SMA)* were analyzed by qRT–PCR in livers from mice that underwent CCl_4_ treatment or mice that underwent BDL for different timepoints. **C** The PCR products from the 5'-RACE procedure and 3'-RACE procedure underwent agarose gel electrophoresis. **D** The nuclei or cytoplasm of single-cell suspensions from the normal liver were isolated, and qRT–PCR analysis detected the expression of *lnc-Helf*, *Neat1*, and *Gapdh*. **E** qRT–PCR was used to assess the expression of *lnc-Helf* in HCs, HSCs, HMs, and LSECs that were isolated from livers of mice with or without CCl_4_ treatment for 6 weeks. **F** qRT–PCR was used to assess the expression of *lnc-Helf* and *Acta2* in the HSCs after culture-induced activation for indicated times. **G** qRT–PCR was used to assess the expression of *lnc-HELF* in liver samples from fibrotic patients (*n* = 28) and healthy people (*n* = 6). Data are presented as mean ± SEM. *p < 0.05, unpaired Student’s *t* test (**A**, **B**, and **E**–**G**)

We previously identified differentially expressed lncRNAs between mouse normal and fibrotic livers [14]. From that study, we noted that lncRNA ENSMUST00000147617 (lnc-Helf) was increased in fibrotic liver (Additional file 1: Fig. S1A). To confirm this result by qRT–PCR, the RNA level of lnc-Helf was detected in fibrotic livers of mice treated with BDL or CCl_4_ for various time periods, and the data revealed that the level of lnc-Helf was markedly increased with persistent injury (Fig. 1A, B). Subsequently, 5′- and 3′-RACE assay in mouse liver demonstrated that lnc-Helf was a 680 nt transcript, consistent with the ensemble database (Fig. 1C; Additional file 1: Fig. S1B). Moreover, sequence homology searches identified a clear lnc-HELF homologous transcript in the human genome with 70% sequence similarity (Additional file 1: Fig. S1C, D). Cell fractionation followed by qRT–PCR and fluorescence in situ hybridization (FISH) assays revealed that lnc-Helf was located both in the nucleus and cytoplasm of primary HSCs and single-cell suspensions from the liver (Fig. 1D; Additional file 1: Fig. S1E, F). As expected, the coding potential calculator 2 (CPC2) and coding potential assessment tool (CPAT) demonstrated that lnc-Helf had no protein coding potential. To assess the expression of lnc-Helf in various cell types of healthy and fibrotic livers, we have isolated HSCs, HCs, HMs, and liver sinusoidal endothelial cells (LSECs) from livers treated with or without CCl_4_ and found that lnc-Helf was mainly expressed in primary HSCs, followed by HMs, rather than HCs and LSECs (Fig. 1E). Notably, lnc-Helf was upregulated in HSCs and HMs of fibrotic livers compared to normal livers (Fig. 1E). However, lnc-Helf was minimally expressed in AML12 cells, LX-2 cells, RAW264.7 cells, human umbilical vein endothelial cells (HUVECs), and mouse BMMs (Additional file 1: Fig. S1G). Moreover, the expression of lnc-Helf was also significantly upregulated at day 7 and day 14 in activated HSCs, compared with day 3 HSCs (Fig. 1F). However, recombinant TGFβ did not regulate the expression of lnc-Helf in primary HSCs (Additional file 1: Fig. S1H). To correlate these findings with data from patients, we tested the RNA level of human lnc-HELF in 6 healthy livers and 28 fibrotic livers, and the data revealed that lnc-HELF was markedly increased in the livers of subjects with fibrosis than it was in individuals with normal livers (Fig. 1G). However, no correlation was found with the level of ACTA2, COL1α1, ALT, and AST (Additional file 1: Fig. S1I–L). Altogether, these data demonstrate that lnc-Helf is increased in mouse and human fibrotic livers, with activated HSCs and HMs.

### Knockdown of lnc-Helf alleviates hepatic fibrosis induced by CCl_4_ and BDL

**Fig. 2:**
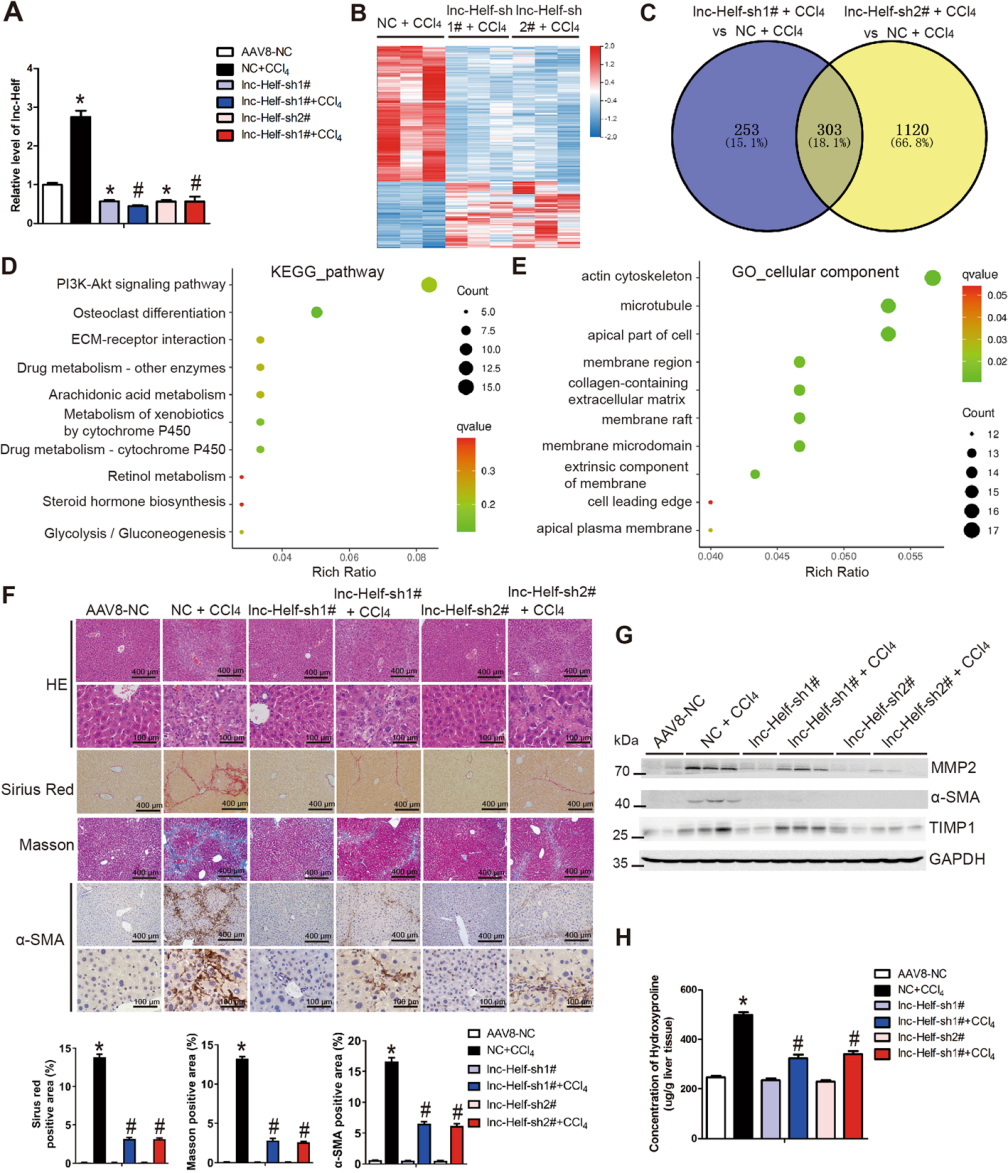
Knockdown of lnc-Helf alleviates hepatic fibrosis induced by CCl_4_. Mice were divided into six groups: AAV8-NC, NC + CCl_4_, lnc-Helf-sh1#, lnc-Helf-sh1# + CCl_4_, lnc-Helf-sh2#, and lnc-Helf-sh2# + CCl_4_. Mice were injected with AAV8-lnc-Helf-shRNAs, or AAV8-NC virus 2 weeks after the first injection of CCl_4_ via tail vein. After CCl_4_ treatment for 8 weeks. **A** qRT–PCR was used to assess the expression of *lnc-Helf* in livers of each group (*n* = 3). **B** The significantly differentially expressed mRNAs were displayed by hierarchical cluster analysis: bright red, upregulation; bright blue, downregulation (*n* = 3). **C**–**E** The Venn diagram, KEGG, and GO analyses of differentially expressed mRNAs in lnc-Helf-sh1# + CCl_4_ mice and lnc-Helf-sh2# + CCl_4_ mice, compared with NC + CCl_4_ mice. **F** The degree of liver fibrosis was evaluated by morphological detection: H&E staining, Masson staining, Sirius red staining, and IHC for α-SMA; five images of each liver and five livers from different mice were quantified for each group; scale bar is 100 μm for 40× and 400 μm for 10× magnifications . **G** Western blot was used to determine the protein level of MMP2, α-SMA, and TIMP1. GAPDH was used as an internal control. **H** The content of hepatic hydroxyproline was quantified in livers of each group (*n* = 5). The data were displayed as hydroxyproline (μg)/liver wet weight. Data are presented as mean ± SEM. ^*/#^*p* < 0.05. *p < 0.05 for AAV8-NC. ^#^*p* < 0.05 for NC + CCl_4_, one-way ANOVA (**A**, **F**, **H**)

To investigate the role of lnc-Helf during liver fibrogenesis in vivo, AAV8-lnc-Helf-shRNA1#, AAV8-lnc-Helf-shRNA2#, or AAV8-NC was injected 2 weeks after the first injection of CCl_4_ via the tail vein. Mice were then treated with CCl_4_ or oil twice per week for an additional 6 weeks. qRT–PCR analysis confirmed that lnc-Helf was knocked down in liver tissues (Fig. 2A). Moreover, we performed RNA-seq to explore the influence of lnc-Helf deficiency on CCl_4_-induced hepatic fibrosis. A total of 556 mRNAs were dysregulated in CCl_4_-treated lnc-Helf-shRNA1# mice, and 1423 mRNAs were dysregulated in CCl_4_-treated lnc-Helf-shRNA2# mice, compared with the CCl_4_-treated AAV8-NC mice (Fig. 2B). Among these, 303 (100 mRNAs were upregulated and 203 mRNAs were downregulated) genes were also dysregulated in the CCl_4_-treated lnc-Helf-shRNAs mice (Fig. 2C; Additional file 1: Fig. S2A, B), and the Kyoto Encyclopedia of Genes and Genomes (KEGG) pathway and Gene Ontology (GO) analyses showed that knockdown of lnc-Helf affected a series of genes related with drug metabolism, arachidonic acid metabolism, retinol metabolism, PI3K-AKT pathway, and ECM–receptor interaction (Fig. 2D, E; Additional file 1: Fig. S2C, D), suggesting that lnc-Helf deficiency regulates CCl_4_-induced liver metabolic dysfunction and fibrosis. To confirm this, we performed hematoxylin and eosin (H&E) staining, Masson staining, Sirius red staining, and immunohistochemistry (IHC) for α-SMA, COL1α1, and TGFβ, and the data revealed that lnc-Helf silencing alleviated hepatic fibrosis induced by CCl_4_ (Fig. 2F and Additional file 1: Fig. S3A). Western blotting demonstrated that lnc-Helf silencing decreased the upregulation of MMP2, α-SMA, and TIMP1 induced by CCl_4_ (Fig. 2G). Consistently, qRT–PCR analysis showed that lnc-Helf deficiency noticeably alleviated CCl_4_-induced upregulation of profibrotic genes (Acta2, Col1α1, Mmp2, Timp1, and Tgfβ1) (Additional file 1: Fig. S3B). The serum level of AST and hepatic hydroxyproline content in CCl_4_-treated lnc-Helf-shRNAs mice were also markedly downregulated compared with CCl_4_-treated AAV8-NC mice (Fig. 2H; Additional file 1: Fig. S3C, D). In addition, CCl_4_-treated lnc-Helf-shRNAs mice exhibited reduced inflammatory response and compensated proliferation compared with CCl_4_-treated AAV8-NC mice, as evidenced by IHC and western blot for CD11b, F4/80, LY6C, TNF-α, IL-1β, MCP1, and PCNA, together with the mRNA level of genes related with inflammation (Tnf-α, Mcp1, Il-6, and Il-1β) and proliferation (Pcna, Ki67 and Cyclin D1) in the livers (Additional file 1: Fig. S4A–D).

**Fig. 3:**
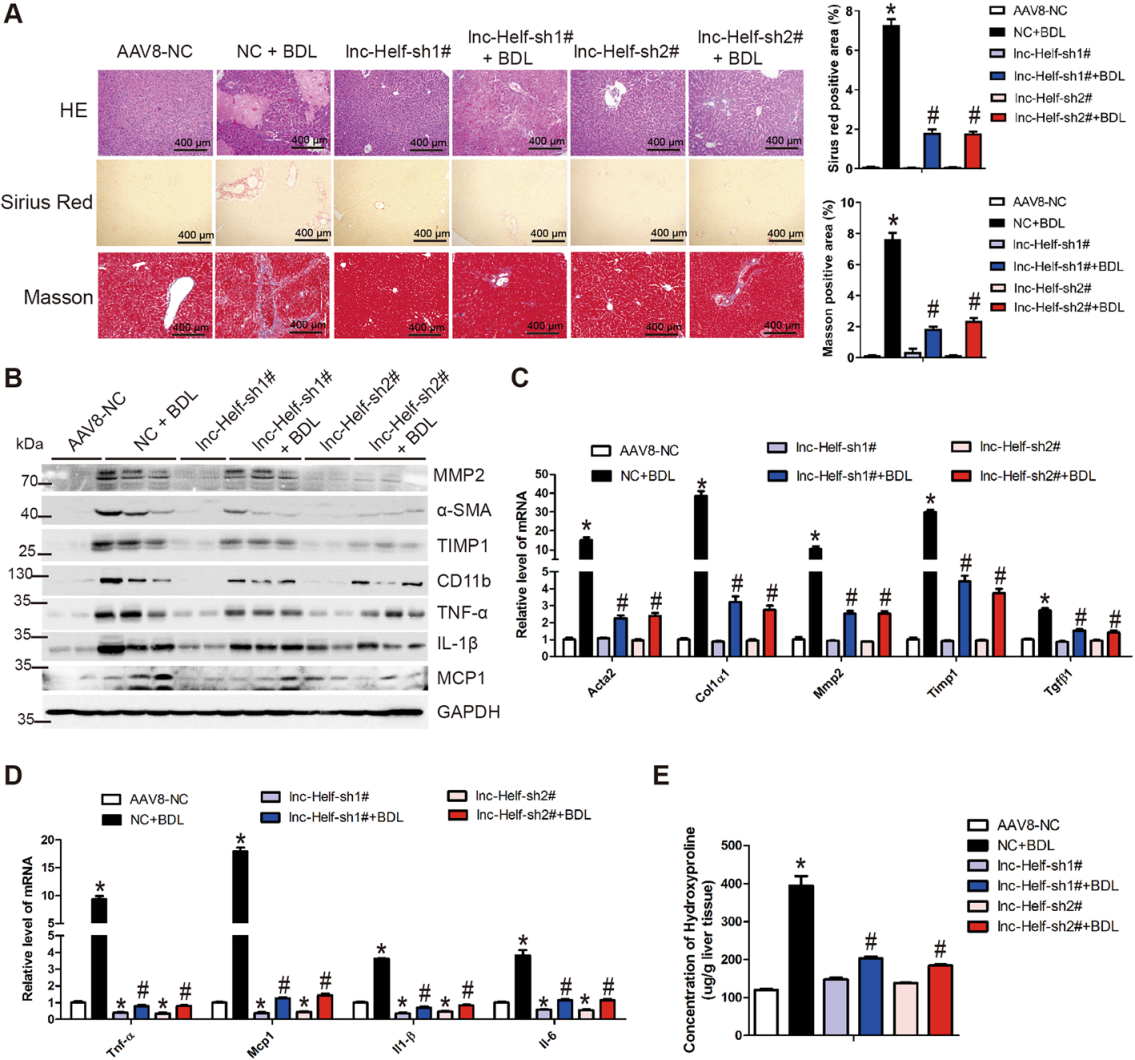
Knockdown of lnc-Helf alleviates hepatic fibrosis induced by BDL. Mice were divided into six groups: AAV8-NC, NC + BDL, lnc-Helf-sh1#, lnc-Helf-sh1# + BDL, lnc-Helf-sh2#, and lnc-Helf-sh2# + BDL. Mice were injected with AAV8-lnc-Helf-shRNAs or AAV8-NC virus 2 days before sham operation or bile duct ligation operation via tail vein. After 21 days of operation. **A** The degree of liver fibrosis was evaluated by morphological detection: H&E staining, Masson staining, and Sirius red staining. Five images of each liver and five livers from different mice were quantified for each group; Scale bar is 400 μm for 10× magnification. **B** Western blot was used to determine the protein level of MMP2, α-SMA, TIMP1, CD11b, TNF-α, IL-1β, and MCP1. GAPDH was used as an internal control. **C**, **D** qRT–PCR was used to assess the expression of profibrogenic genes (*Acta2*, *Col1α1*, *Mmp2*, *Timp1*, and *Tgfβ1*) and proinflammatory genes (*Tnf-α*, *Mcp1*, *Il-6*, and *Il-1β*) in livers of each group (*n* = 3). **E** The content of hepatic hydroxyproline was quantified in livers of each group (*n* = 5). The data were displayed as hydroxyproline (μg)/liver wet weight. Data are presented as mean ± SEM. ^*/#^*p* < 0.05. *p < 0.05 for AAV8-NC. ^#^*p* < 0.05 for NC + BDL, one-way ANOVA (**A** and **C**–**E**)

The results were further confirmed in BDL-induced hepatic fibrosis model. As shown in Fig. 3 and Additional file 1: Fig. S5, the BDL group mice developed serious hepatic fibrosis, while lnc-Helf silencing alleviated BDL-induced hepatic fibrosis and inflammation as demonstrated by H&E staining, Masson staining, Sirius red staining, serum ALT, AST level, liver hydroxyproline content, western blot, and qRT–PCR (Fig. 3A–E; Additional file 1: Fig. S5A–D). Altogether, these data prove that knockdown of lnc-Helf obviously alleviates hepatic inflammation and fibrosis induced by CCl_4_ and BDL.

### lnc-Helf promotes HSCs activation and proliferation

**Fig. 4:**
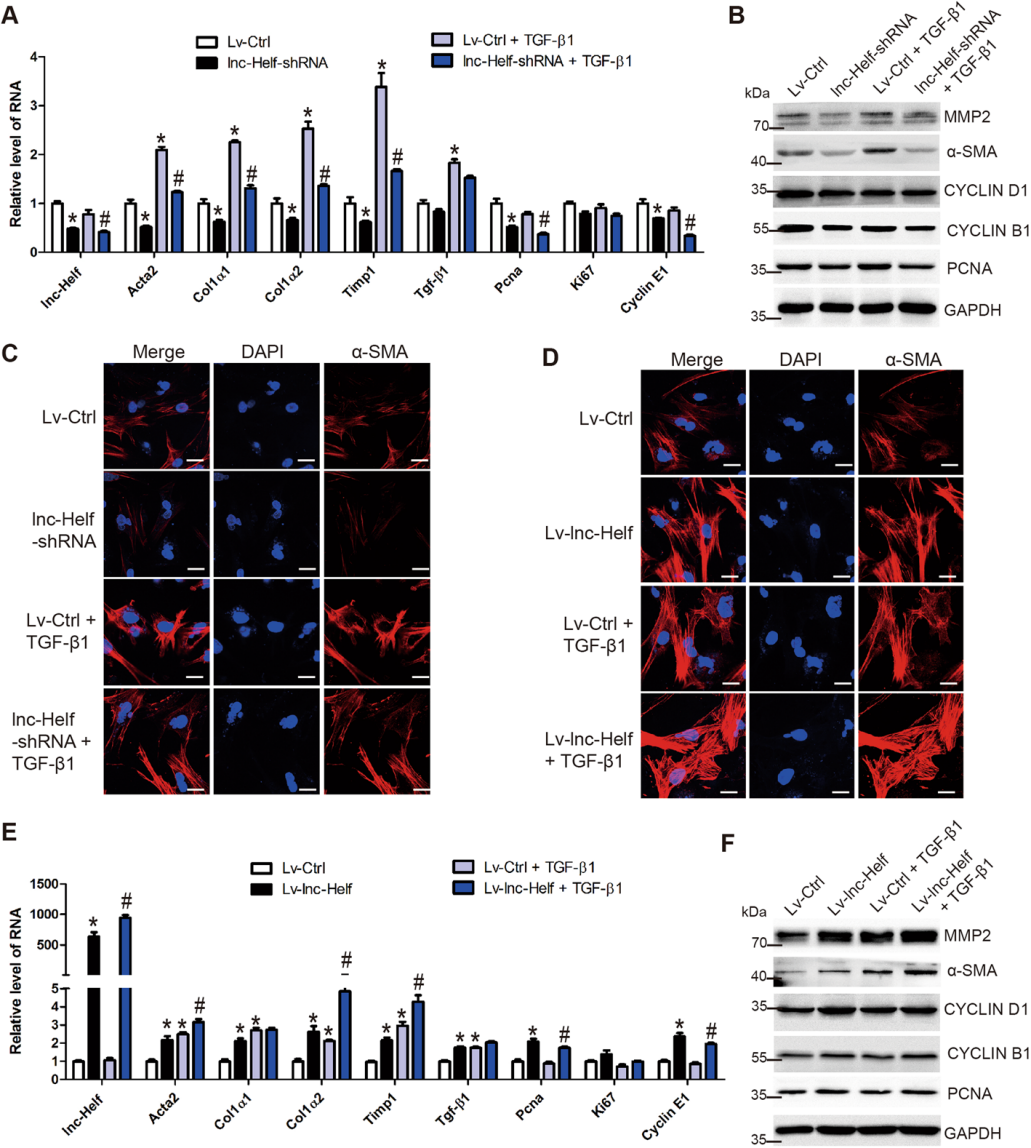
lnc-Helf promotes HSCs activation and proliferation. **A**–**C** Primary HSCs were isolated and infected with lnc-Helf-shRNA or lenti-control at day 2 for 48 h, following the treatment with 10 ng/ml TGF-β1 for 24 h. qRT-PCR was used to assess the RNA level of *lnc-Helf*, *Acta2*, *Col1α1*, *Col1α2*, *Timp1*, *Tgf-β1*, *Pcna*, *Ki67*, and *Cyclin E1* (**A**); western blot was used to determine the expression of α-SMA, MMP2, CYCLIN D1, CYCLIN B1, and PCNA (**B**). GAPDH was used as an internal control; the expression and location of α-SMA was assessed by confocal microscopy (**C**). Scale bar is 20 μm. **D**–**F** Primary HSCs were isolated and infected with lenti-lnc-Helf or lenti-control at day 2 for 48 h, following the treatment with 10 ng/ml TGF-β1 for 24 h. The expression and location of α-SMA was assessed by confocal microscopy (**D**). Scale bar is 20 μm. qRT–PCR was used to detect the RNA level of *lnc-Helf*, *Acta2*, *Col1α1*, *Col1α2*, *Timp1*, *Tgf-β1*, *Pcna*, *Ki67*, and *Cyclin E1* (**E**); western blot was used to determine the expression of α-SMA, MMP2, CYCLIN D1, CYCLIN B1, and PCNA (**F**). GAPDH was used as an internal control. Data are presented as mean ± SEM. ^*/#^*p* < 0.05. *p < 0.05 for LV-control. ^#^*p* < 0.05 for LV-control + TGF-β1, one-way ANOVA (**A**, **E**)

Activated HSCs are the central ECM-producing cells in the liver [3]. To investigate the effect of lnc-Helf on the activation and proliferation of HSCs, lnc-Helf was knocked down by lentivirus vector of lnc-Helf-shRNA at day 2 primary HSCs. Cells were treated with recombinant TGFβ for 24 h, and qRT–PCR analysis detected the expression of fibrosis-associated (Acta2, Col1α1, Col1α2, Timp1, and Tgf-β1) and proliferation-associated genes (Pcna, Ki67, and Cyclin E1). We found that knockdown of lnc-Helf significantly reduced the level of lnc-Helf, profibrotic, and proproliferation genes in the presence or absence of TGFβ (Fig. 4A). Consistently, western blot showed that lnc-Helf silencing downregulated the protein level of α-SMA, MMP2, CYCLIN B1, and PCNA in the presence or absence of TGFβ (Fig. 4B; Additional file 1: Fig. S6A). Similar results were also obtained by confocal microscopy (Fig. 4C; Additional file 1: Fig. S6B). On the other hand, forced expression of lnc-Helf increased the level of the profibrotic and proproliferation genes evaluated by western blot, qRT–PCR, and confocal microscopy (Fig. 4D–F and Additional file 1: Fig. S6C, D). To further confirm the results in LX-2 cells, lnc-HELF was overexpressed in the cells by lentivirus vector of lnc-HELF (LV-lnc-HELF), as the level of lnc-HELF in LX-2 cells was quite low. qRT–PCR and western blot analysis revealed that overexpression of lnc-HELF enhanced the level of the profibrotic and proproliferation genes (Additional file 1: Fig. S6E, F). In addition, CCK8 assays demonstrated that overexpression of lnc-HELF promoted LX-2 cells and primary HSCs proliferation (Additional file 1: Fig. S6G, H). Altogether, these data suggest that lnc-Helf accelerates HSCs activation and proliferation.

### lnc-Helf induces HMs M1 polarization and proliferation

**Fig. 5:**
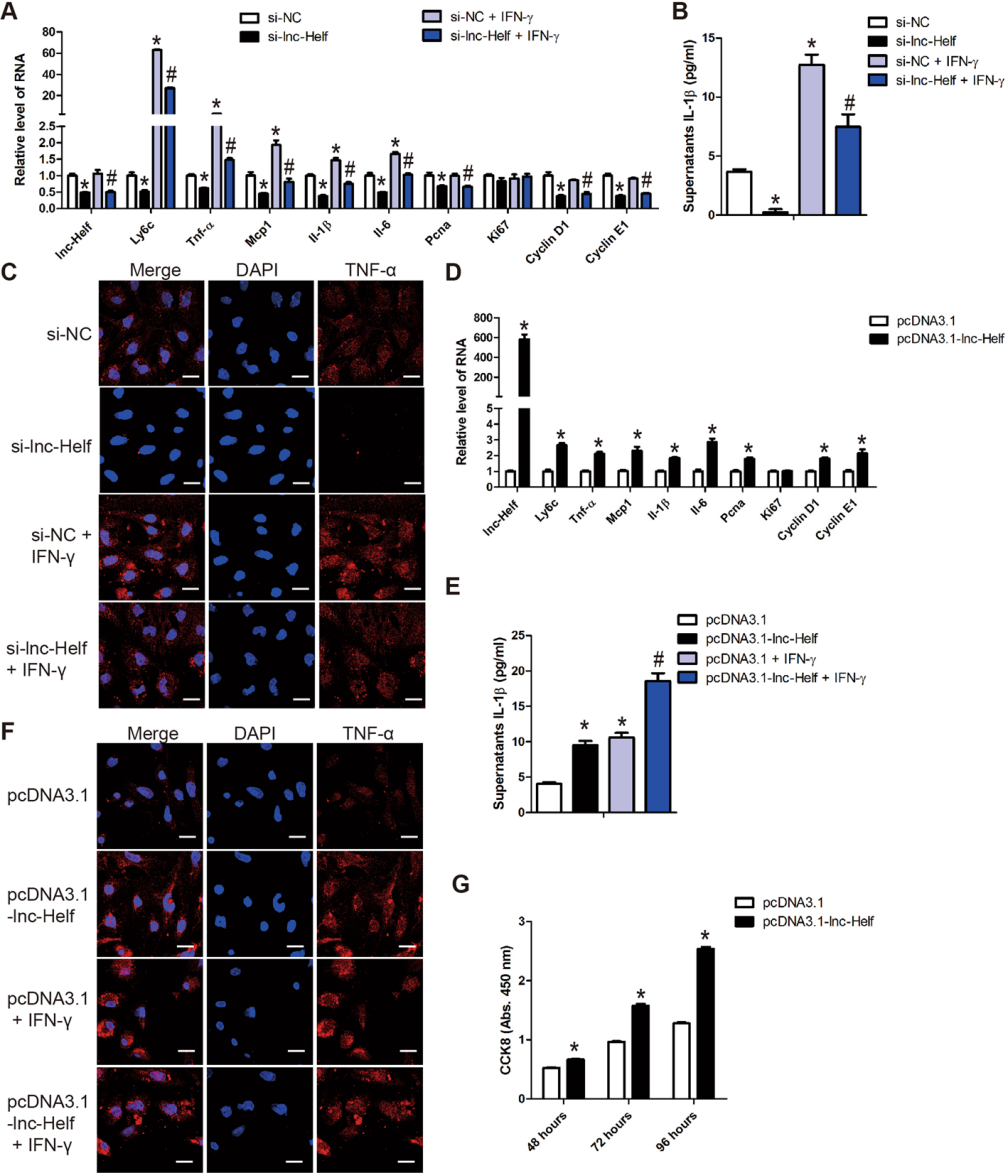
lnc-Helf promotes HMs M1 polarization and proliferation. **A**–**C** Mouse primary HMs transfected with siRNA for 24 h, following treatment with 20 ng/ml IFN-γ for 24 h. qRT–PCR analysis was used to detect the RNA level of *lnc-Helf*, *Ly6c*, *Tnf-α*, *Mcp-1*, *Il-1β*, *Il-6*, *Pcna*, *Cyclin D1*, and *Cyclin E1* (**A**); mature supernatant IL-1β level was detected by ELISA (**B**); the expression and location of TNF-α was assessed by confocal microscopy (**C**). Scale bar is 20 μm. **D** HMs were transfected with pcDNA3.1-lnc-Helf or pcDNA3.1 for 48 h; qRT–PCR was used to assess the expression of *lnc-Helf*, *Ly6c*, *Tnf-α*, *Mcp-1*, *Il-1β*, *Il-6*, *Pcna*, *Cyclin D1*, and *Cyclin E1*. **E**, **F** HMs were transfected with pcDNA3.1-lnc-Helf or pcDNA3.1 for 48 h, following treatment with 20 ng/ml IFN-γ for 24 h. Mature supernatant IL-1β level was detected by ELISA (**E**); the expression and location of TNF-α was assessed by confocal microscopy (**F**). Scale bar is 20 μm. **G** RAW264.7 cells were transfected with pcDNA3.1-lnc-Helf or pcDNA3.1 for the indicated times, cell proliferation was detected by CCK8. Data are presented as mean ± SEM. ^*/#^*p* < 0.05. *p < 0.05 for si-NC or pcDNA3.1. ^#^*p* < 0.05 for si-NC + IFN-γ or pcDNA3.1 + IFN-γ, one-way ANOVA (**A**, **B**, **E**) and unpaired Student’s *t* test (**D**, **G**)

Given the evidences that lnc-Helf was upregulated in HMs from the fibrotic livers and lnc-Helf silencing ameliorated CCl_4_- and BDL- induced hepatic inflammation in vivo, we investigated the role of lnc-Helf on the polarization and proliferation of macrophage in vitro. Mouse HMs were transfected with lnc-Helf siRNA following a treatment with 20 ng/ml IFN-γ. qRT–PCR analysis showed that lnc-Helf silencing decreased the expression of lnc-Helf, proinflammatory genes including Ly6c, Tnf-α, Il-6, Mcp-1, and Il-1β, as well as proproliferation genes including Pcna, Cyclin D1, and Cyclin E1 in the absence or presence of IFN-γ (Fig. 5A). Moreover, mature IL-1β level in the supernatant was also assessed by enzyme-linked immunosorbent assay (ELISA), and the data indicated that IL-1β level was downregulated in the supernatant of lnc-Helf-silenced HMs compared with the control (Fig. 5B). Similarly, confocal microscopy revealed that lnc-Helf silencing decreased the expression of TNF-α (Fig. 5C). On the other hand, qRT–PCR analysis revealed that lnc-Helf silencing promoted, while forced expression of lnc-Helf decreased the expression of anti-inflammatory genes including Mrc1, Cd163, and Il10 (Additional file 1: Fig. S7A, B). In addition, forced expression of lnc-Helf increased the level of proinflammatory and proproliferation genes evaluated by qRT–PCR, ELISA, and confocal microscopy (Fig. 5D–F). Consistently, similar results were also obtained in RAW264.7 cells (Additional file 1: Fig. S7C, D). In addition, CCK8 assay demonstrated that overexpression of lnc-Helf markedly promoted RAW264.7 cells proliferation (Fig. 5G). Altogether, these data demonstrate that lnc-Helf induces the M1 polarization and proliferation of macrophages.

### lnc-Helf promotes hepatic inflammation and fibrosis through activating AKT pathway

**Fig. 6:**
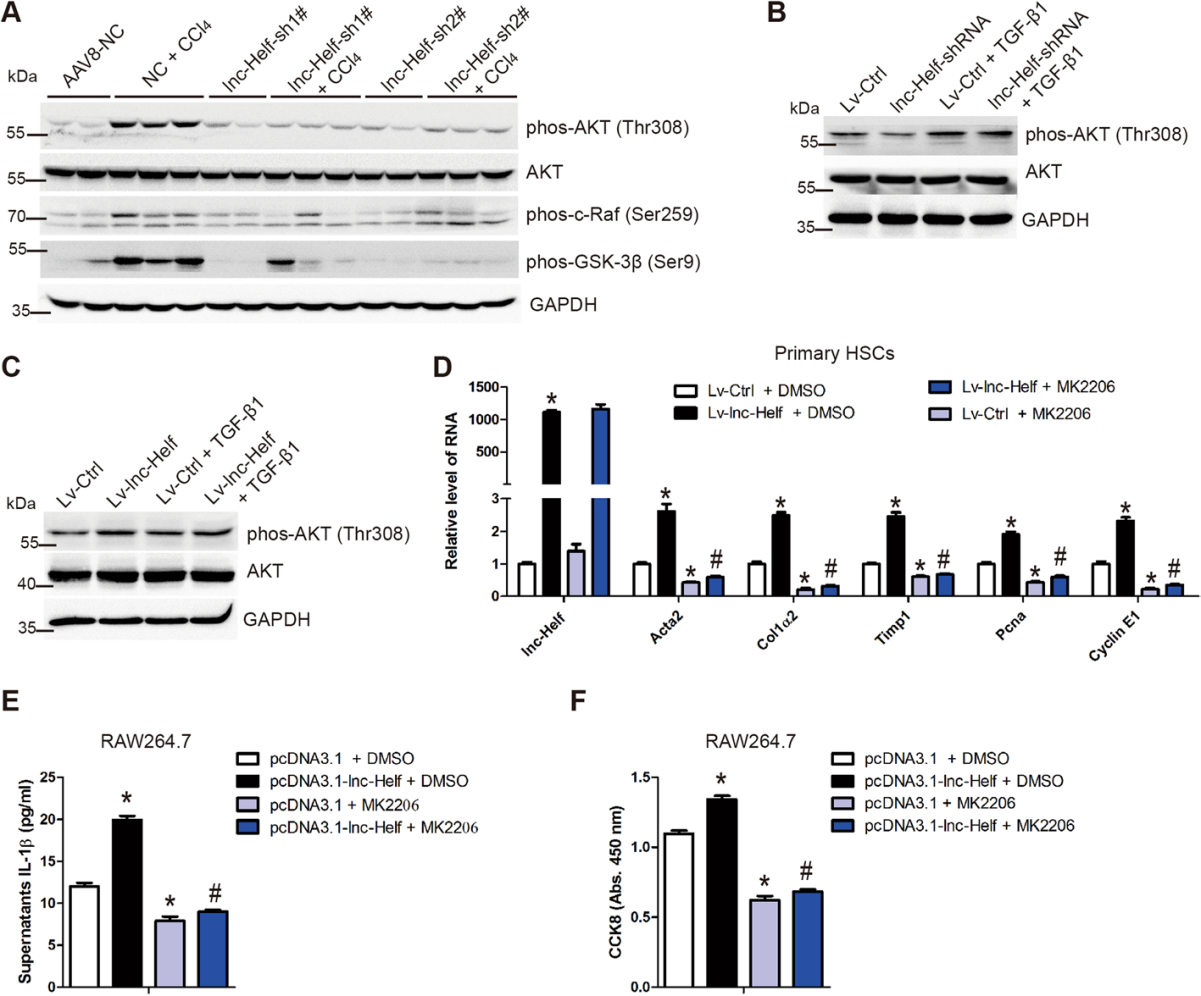
lnc-Helf promotes hepatic inflammation and fibrosis through activating AKT pathway. **A** Mice were divided into six groups: AAV8-NC, NC + CCl_4_, lnc-Helf-sh1#, lnc-Helf-sh1# + CCl_4_, lnc-Helf-sh2#, and lnc-Helf-sh2# + CCl_4_. Western blot was used to determine the protein level of phos-AKT (Thr308), phos-c-Raf1 (Ser259), phos-GSK-3β (Ser9), and AKT in liver tissues of each group. **B**, **C** Primary HSCs at day 2 were infected with lenti-control, lnc-Helf-shRNA, or lenti-lnc-Helf for 48 h, following treatment with 10 ng/ml TGF-β1 for 24 h. Western blot was used to determine the protein level of phos-AKT (Thr308) and AKT. GAPDH was used as an internal control. **D** Primary HSCs treated with or without the AKT inhibitor MK2206 at day 2 were infected with lenti-control or lenti-lnc-Helf for 72 h. qRT–PCR was used to detect the RNA level of *lnc-Helf*, *Acta2*, *Col1α2*, *Timp1*, *Pcna*, and *Cyclin E1*. **E**, **F** RAW264.7 cells treated with or without the AKT inhibitor MK2206 were transfected with pcDNA3.1-lnc-Helf or pcDNA3.1 for 72 h. Mature supernatant IL-1β level was detected by ELISA (**E**); cell proliferation was detected by CCK8 (**F**). Data are presented as mean ± SEM. ^*/#^*p* < 0.05. *p < 0.05 for LV-control + DMSO or pcDNA3.1 + DMSO. ^#^*p* < 0.05 for LV-lnc-Helf + DMSO or pcDNA3.1-lnc-Helf + DMSO, one-way ANOVA (**D**–**F**)

Since the KEGG pathway and GO analyses indicated that knockdown of lnc-Helf affected a serial of genes related with the PI3K-AKT pathway (Fig. 2D), we measured the level of phos-AKT (Thr308), phos-c-Raf1 (Ser259), phos-GSK-3β (Ser9), and AKT in liver tissues from lnc-Helf-silenced or control mice treated with or without CCl_4_/BDL. Western blot analysis revealed that knockdown of lnc-Helf suppressed CCl_4_- and BDL-induced phosphorylation of AKT and GSK-3β (Fig. 6A and Additional file 1: Fig. S8A). Moreover, knockdown of lnc-Helf reduced, while overexpression of lnc-Helf promoted, the phosphorylation of AKT in HSCs and LX-2 cells (Fig. 6B, C; and Additional file 1: Fig. S8B), suggesting that lnc-Helf silencing alleviated hepatic inflammation and fibrosis through the AKT pathway. Subsequently, MK2206, the specific inhibitor of AKT, was applied in lnc-Helf-upregulated HSCs and RAW264.7 cells. qRT–PCR analysis showed that MK2206 abrogated lnc-Helf overexpression and increased the expression of profibrotic, proinflammatory, and proproliferation genes (Fig. 6D; Additional file 1: Fig. S8C). Consistently, ELISA and CCK8 assays demonstrated that the increased supernatant IL-1β levels and cell proliferation by lnc-Helf overexpression was abrogated by MK2206 (Fig. 6E, F). Altogether, these data indicate that lnc-Helf promotes hepatic inflammation and fibrosis through activating AKT pathway.

### lnc-Helf promotes hepatic inflammation and fibrosis by interacting with PTBP1 to facilitate PIK3R5 mRNA stabilization

**Fig. 7:**
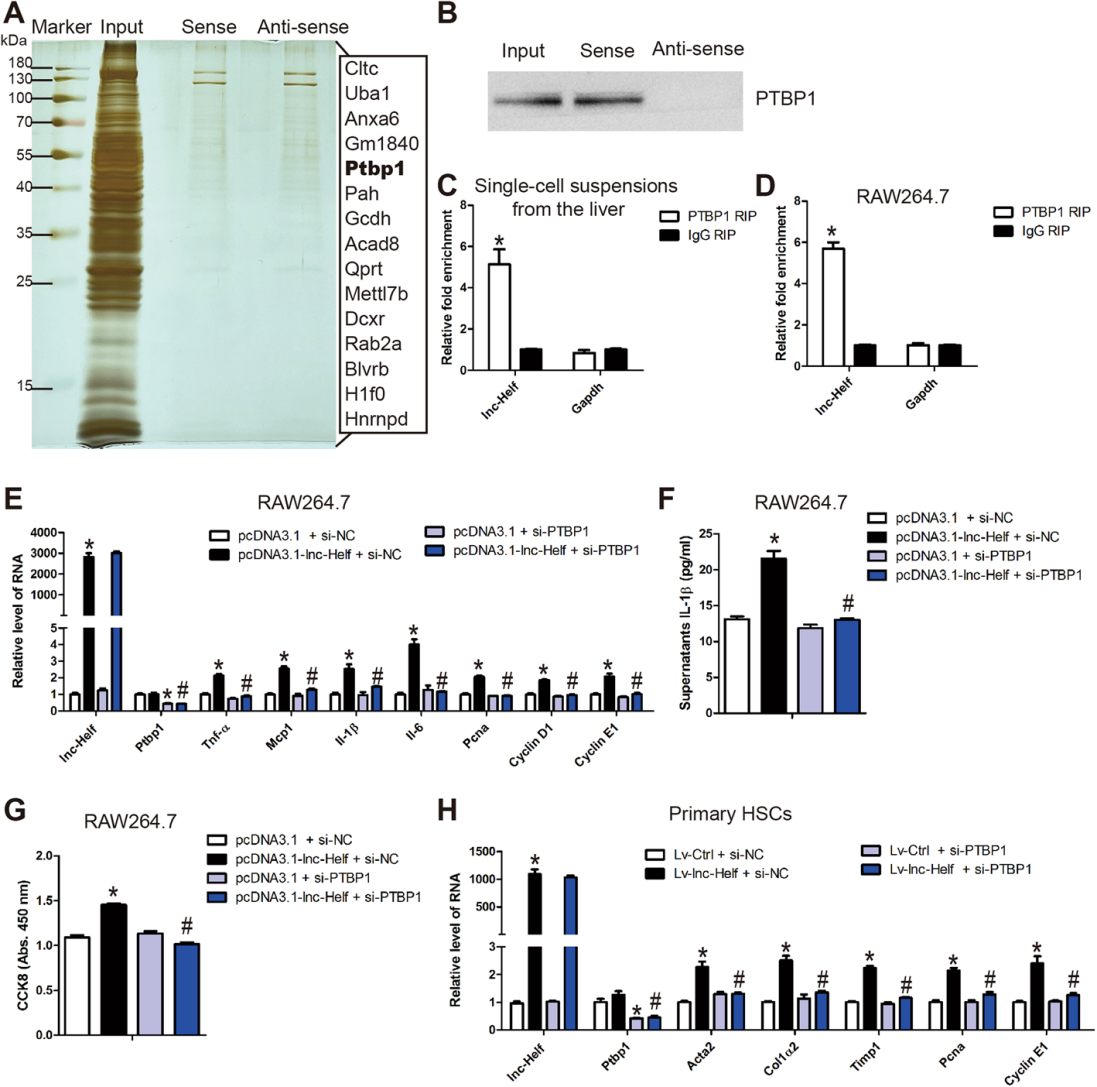
lnc-Helf promotes hepatic inflammation and fibrosis by interacting with PTBP1. **A** In vitro RNA pulldown–mass spectrometry assay in single liver cell lysates. **B** The specific interaction of sense lnc-Helf RNA with PTBP1 protein was determined by western blot. **C**, **D** RIP–qRT–PCR assay in single-cell suspensions from the liver (**C**) and RAW264.7 cells (**D**). **E** qRT–PCR was used to detect the expression of *lnc-Helf*, *Ptbp1*, *Tnf-α*, *Mcp-1*, *Il-1β*, *Il-6*, *Pcna*, *Cyclin D1*, and *Cyclin E1* in lnc-Helf-overexpressed RAW264.7 cells simultaneously transfected with siPTBP1. **F** Mature supernatant IL-1β levels in lnc-Helf-overexpressed RAW264.7 cells simultaneously transfected with siPTBP1 was detected by ELISA. **G** Cell proliferation of lnc-Helf-overexpressed RAW264.7 cells simultaneously transfected with siPTBP1 was detected by CCK8. **H** The expression of *lnc-Helf*, *Ptbp1*, *Acta2*, *Col1α2*, *Timp1*, *Pcna*, and *Cyclin E* in lnc-Helf-upregulated primary HSCs, simultaneously transfected with siPTBP1, was detected by qRT–PCR. Data are presented as mean ± SEM. ^*/#^*p* < 0.05. *p < 0.05 for IgG RIP or pcDNA3.1 + si-NC or LV-control + si-NC. ^#^*p* < 0.05 for pcDNA3.1-lnc-Helf + si-NC or LV-lnc-Helf + si-NC, unpaired Student’s *t* test (**C**, **D**) and one-way ANOVA (**E**–**H**)

To further explore the molecular mechanism of lnc-Helf during hepatic fibrogenesis, we performed RNA pulldown–mass spectrometry (MS) assay to identify the proteins binding with lnc-Helf (Fig. 7A; Additional file 1: Table S2). Among the lnc-Helf-bound proteins, PTBP1, which is an RNA-binding protein and regulates pre-mRNA splicing and mRNA stability, caught our attention. Western blot further confirmed that lnc-Helf interacted with PTBP1 (Fig. 7B). Moreover, lnc-Helf was detected in the immunoprecipitates of PTBP1 by RIP assay (Fig. 7C, D), further demonstrating the binding of lnc-Helf to PTBP1. Subsequently, specific siRNAs targeting PTBP1 were transfected into lnc-Helf-overexpressed RAW264.7 cells, and the data of the qRT–PCR, ELISA and CCK8 demonstrated that PTBP1 silencing blocked lnc-Helf overexpression-induced inflammation response and proliferation (Fig. 7E–G). Consistently, these data were also confirmed in HSCs (Fig. 7H; Additional file 1: Fig. S9). All these data suggest that lnc-Helf aggravates hepatic inflammation and fibrosis via binding with PTBP1.

**Fig. 8:**
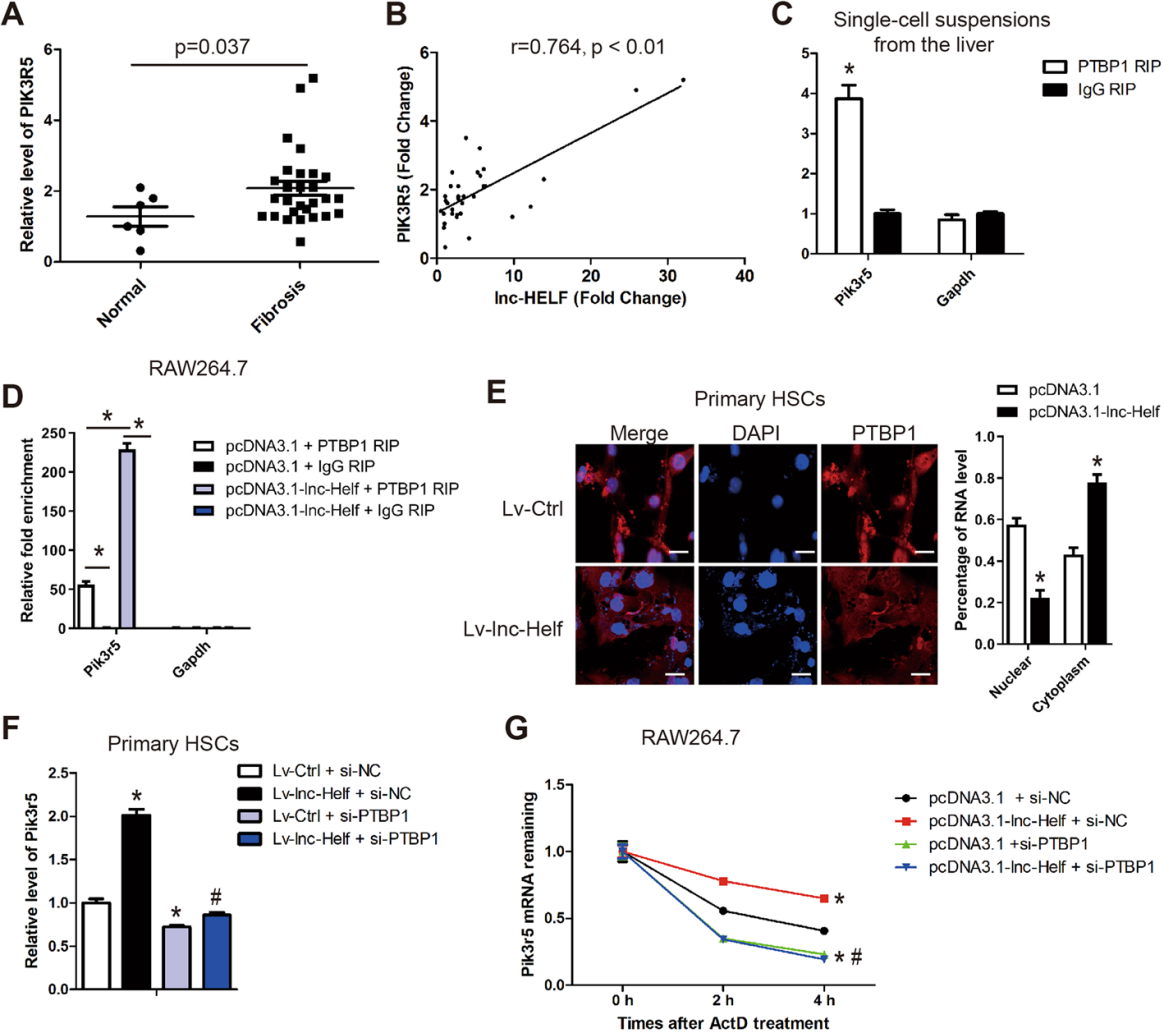
lnc-Helf promotes hepatic inflammation and fibrosis by interacting with PTBP1 to facilitate PIK3R5 mRNA stabilization. **A** The RNA level of *PIK3R5* was detected in liver samples of fibrotic patients (*n* = 28) and healthy people (*n* = 6) by qRT–PCR analysis. **B** The correlation of *lnc-HELF* and *PIK3R5* was assessed by Pearson correlation analysis, *n* = 34. **C**, **D** RIP–qRT–PCR assay in single-cell suspensions from the liver (**C**) and RAW264.7 cells transfected with pcDNA3.1-lnc-Helf or pcDNA3.1 (**D**). **E** The expression and location of PTBP1 in primary HSCs infected with lenti-control or lenti-lnc-Helf was assessed by confocal microscopy. Scale bar is 20 μm. The fluorescence intensity of nuclear and cytoplasm was quantified on the right. **F** qRT–PCR was used to detect the RNA level of *Pik3r5* in lnc-Helf-overexpressed primary HSCs simultaneously transfected with siPTBP1. **G** RAW264.7 cells transfected with pcDNA3.1 or pcDNA3.1-lnc-Helf with or without si-PTBP1 were treated with 5 μg/ml actinomycin D. RNA of each group was extracted at indicated time points (0, 2, 4 h) and the expression of *Pik3r5* mRNA was analyzed by qRT–PCR and normalized to *Gapdh*. Data are presented as mean ± SEM. ^*/#^*p* < 0.05. *p < 0.05 for IgG RIP or LV-control + si-NC or pcDNA3.1 + si-NC. ^#^*p* < 0.05 for LV-lnc-Helf + si-NC or pcDNA3.1-lnc-Helf + si-NC, unpaired Student’s *t* test (**A**, **C**, **E**) and one-way ANOVA (**D**, **F**, **G**)

PTBP1 belongs to a family of RNA-binding proteins famous for its function in regulating mRNA stability [20, 21]. Therefore, we assumed that the interaction between lnc-Helf and PTBP1 may regulate the effect of PTBP1 on its target mRNAs, thus activating the AKT pathway. The online bioinformatics analysis tools (RPIseq, RBPmap) were used to predict the target RNAs binding with PTBP1 (Additional file 1: Fig. S10A, B). PIK3R5, which was significantly decreased in CCl_4_-treated lnc-Helf-shRNAs mice compared with CCl_4_-treated AAV8-NC mice (Fig. 2B, C; Additional file 1: Fig. S2A, B), was predicted to comprise several PTBP1 binding sites. We first measured the expression of PIK3R5 and PTBP1 in 6 healthy liver tissues and 28 fibrotic liver tissues, and the results revealed that PIK3R5 rather than PTBP1 was increased in fibrotic livers (Fig. 8A; Additional file 1: Fig. S10C). Moreover, lnc-HELF was found to be positively correlated with PIK3R5 and PTBP1 (Fig. 8B; Additional file 1: Fig. S10D). A correlation of PTBP1 with PIK3R5 was also observed (Additional file 1: Fig. S10E). Furthermore, the expression of Pik3r5 but not Ptbp1 was upregulated in mouse fibrotic livers, induced by CCl_4_ and BDL, and deficiency of lnc-Helf abrogated this upregulation (Additional file 1: Fig. S10F–I). In addition, knockdown of lnc-Helf reduced the level of Pik3r5, while forced expression of lnc-Helf promoted the level of Pik3r5, in primary HSCs, LX-2 cells, primary HMs ,and RAW264.7 cells (Additional file 1: Fig. S11A–F). Next, RIP assay demonstrated that Pik3r5 mRNA was enriched by PTBP1 in single-cell suspensions from the liver (Fig. 8C) and RAW264.7 cells, and this enrichment was markedly augmented by lnc-Helf upregulation (Fig. 8D), suggesting that interaction of lnc-Helf and PTBP1 accelerated PTBP1 binding to Pik3r5 mRNA. Interestingly, confocal microscopy demonstrated that forced expression of lnc-Helf promoted PTBP1 to translocate into the cytoplasm (Fig. 8E). Indeed, qRT–PCR analysis demonstrated that knockdown of PTBP1 blocked lnc-Helf overexpression-induced upregulation of Pik3r5 in HSCs and RAW264.7 cells (Fig. 8F; Additional file 1: Fig. S11G). Then we analyzed how PTBP1 regulates Pik3r5 expression. Actinomycin D, which blocks de novo transcription, was used in RAW264.7 cells transfected with PTBP1-siRNA or pcDNA3.1-lnc-Helf, alone or in combination. The results demonstrated that overexpression of lnc-Helf decreased Pik3r5 mRNA degradation via PTBP1 (Fig. 8G). Altogether, the data reveal that lnc-Helf aggravates hepatic inflammation and fibrosis by interacting with PTBP1 to facilitate PIK3R5 mRNA stabilization.

## Discussion

Cirrhosis is the progressive stage of hepatic fibrosis, which leads by the unbalanced and dynamic ECM remodeling process due to persistent liver injury induced by multiple chronic liver diseases [1, 2]. It is well accepted that the central feature of fibrogenesis is HSC activation, which is caused by multiple inducements, including cytokines, chemokines, damage-associated molecular patterns, and other mediators secreted by liver non-parenchymal cells, such as HMs [1, 3]. Upon liver injury, HMs are activated and release various cytokines and chemokines to perpetuate hepatic inflammation, which is a central point in the progression or resolution of hepatic fibrosis [5]. Continuous progress has been made during the last thirty years in exploring the cellular and molecular mechanisms of hepatic fibrosis. However, the prevalence of liver cirrhosis is still surging and no specific drugs have been approved [2]. Therefore, the further understanding of the molecular mechanisms participate in HSCs and HMs activation during liver fibrosis can provide experimental evidence in developing a promising antifibrotic approach. In our study, a novel lnc-Helf was identified that was increased in human and mouse fibrotic liver tissues, as well as in the HSCs and HMs from fibrotic livers of mice. However, lnc-Helf was barely expressed in BMMs and RAW264.7 cells, suggesting that illustrating the exact function of lnc-Helf in the liver may have the potential to elucidate a specific target for hepatic fibrosis. Furthermore, the in vivo experiments demonstrated that knockdown of lnc-Helf by AAV8 vector reduced hepatic inflammation and fibrosis induced by CCl_4_ and BDL. The in vitro data showed that lnc-Helf silencing markedly inhibited the activation and proliferation of HSCs in the presence or absence of TGFβ. Additionally, lnc-Helf promoted the M1 polarization and proliferation of macrophages, whether IFN-γ was present or absent. Unfortunately, our data revealed that lnc-Helf did not mediate TGFβ-induced HSC activation and IFN-γ-induced HM M1 polarization. All these results demonstrate that lnc-Helf plays a central role in regulating the activation and proliferation of HSCs and HMs, thus providing a possible therapeutic strategy for hepatic fibrosis.

Many molecules and pathways have been documented to induce the activation and proliferation of HSCs as well as the M1 polarization of HMs in the pathogenesis of hepatic fibrosis, including TGFβ, PI3K/AKT, NFκB, and MAPK pathways [1, 14–17]. Notably, PI3K/AKT signaling is a highly conserved pathway that regulates diverse cellular functions, including autophagy, differentiation, apoptosis, and proliferation [22]. PI3Ks, which consist of class I, class II, and class III, contain intracellular lipid kinase activity. Among these classes, class I PI3K, consisting of a catalytic p110 subunit and a regulatory subunit, played an important function in cell proliferation. p85 and p101 (PIK3R5) or p84/p87 are the regulatory subunits of class IA and class IB PI3K, respectively [22, 23]. Upon stimulation, PI3K is transferred to the cell membrane and activates numerous proteins by generating phosphatidylinositol-3,4,5-trisphosphate to recruit target proteins such as AKT, which initiates downstream substrates that regulate cellular activities [22, 23]. Cumulative evidence suggests that the PI3K/AKT pathway is highly correlated with HSC activation and proliferation [24–27]. Several studies have revealed that inhibiting PI3K reduces HSC proliferation and ECM-related gene expression through blocking downstream effectors, such as AKT [24, 27]. On the other hand, PI3K/AKT signaling related to macrophage polarization is reported to regulate tremendous pathways, including NF-κB and MAPK signaling. However, the PI3K/AKT pathway plays a promotive or inhibitive role in regulating inflammatory responses [28]. PI3K has been reported to activate NF-κB, which is downstream of AKT, thus promoting proinflammatory cytokine secretion [23, 29]. Other studies have revealed that the PI3K inhibitor stimulates macrophage M2 polarization [30]. Moreover, AKT has opposite effects in the regulation of macrophage polarization, AKT1 deletion promotes M1 macrophages, whereas AKT2 ablation prevents the M1 phenotype [31]. All these studies show that the exact functions of PI3K/AKT signaling in the regulation of inflammatory response during fibrogenesis are still not clearly clarified and need further exploration. In this study, our results revealed that silencing of lnc-Helf reduced CCl_4_- and BDL-induced phosphorylation of AKT, and the specific inhibitors of AKT abrogated lnc-Helf overexpression-induced upregulation of profibrotic, proinflammatory, and proproliferation genes, which suggest that the PI3K/AKT pathway functions as a positive effector of inflammatory response during fibrogenesis and that lnc-Helf promotes hepatic inflammation and fibrosis through activating AKT pathway.

Emerging evidence has indicated that lncRNAs play a vital role in various pathophysiological processes via controlling gene expression based on cell location [7, 32–35]. In the nucleus, lncRNAs are involved in alternative splicing, participate in transcriptional regulation, and regulate genes’ epigenetic state. In the cytoplasm, lncRNAs control gene expression via altering mRNAs stability, affecting mRNAs translation efficacy, and function as precursors of miRNAs or ceRNA [7]. In this study, our data demonstrated that lnc-Helf was located in the nucleus and cytoplasm of HSCs and single-cell suspensions from the liver. Importantly, it is now widely understood that lncRNAs may alter mRNA stability or translation efficacy via serving as scaffolds for RNA-binding proteins [20, 21, 36]. To investigate the mechanism of lnc-Helf on hepatic fibrosis, we conducted RNA pulldown–MS analysis to identify the proteins that interact with lnc-Helf (Additional file 2). Our results demonstrated that PTBP1, an RNA-binding protein that regulates almost all steps of mRNA post-transcription expression, including mRNA transport and localization, alternative splicing, translation initiation, and mRNA stability [20, 21, 36–38], bound with lnc-Helf. It has been reported that PTBP1 interacts with diverse lncRNAs and is implicated in various liver diseases [20, 21, 38]. For instance, H19 promotes the expression of let-7 via suppressing PTBP1 binding to the let-7 precursors in the process of cholestatic hepatic fibrosis [38]. Another study also demonstrated that H19 increased the interaction between PTBP1 and SREBP1c mRNA, thus facilitating stability and transcription [20]. Moreover, it has been reported that MEG3 causes cholestatic hepatic injury by promoting Shp mRNA degradation through recruiting PTBP1 to its mRNA [21]. In this study, we found that lnc-Helf bound with PTBP1 and promoted its translocation into the cytoplasm, thus facilitating its interaction with Pik3r5 mRNA, resulting in increased stability and AKT pathway activation, which is consistent with the previous study where PTBP1 promoted the progression of breast cancer via the AKT pathway [39].

## Conclusion

Our data unveils a lnc-Helf/PTBP1/PIK3R5/AKT feedforward amplifying signaling to exacerbate the process of hepatic fibrosis and inflammation, thus providing a possible therapeutic strategy for hepatic fibrosis (Additional file 1: Fig. S12).

### Supplementary Information

**Additional file 1: Fig. S1, related to Fig. S1.** (A) The fold change of *lnc-Helf* was shown according to the microarray data. (B, C) The nucleotide sequence of mouse *lnc-Helf* and human *lnc-HELF*. (D) The comparison between human and mouse *lnc-Helf* sequences. (E) The nuclei or cytoplasm of primary HSCs was isolated, and qRT–PCR analysis detected the expression of *lnc-Helf*, *Neat1*, and *Gapdh*. (F) The intracellular localization of *lnc-Helf* in primary HSCs was measured by RNA-FISH assays; scale bar is 10 μm. (G) qRT–PCR analysis of *lnc-Helf* in AML12 cells, LX-2 cells, RAW264.7 cells, BMMs, and HUVECs (H) qRT–PCR was used to assess the expression of *lnc-Helf* and *Acta2* in primary HSCs cultured at day 2 treated with 10 ng/ml TGF-β for 24 h. (I–L) The correlations of lnc-HELF, ACTA2, COL1α1, ALT, and AST were assessed by Pearson correlation analysis, *n* = 34. *p < 0.05, unpaired Student’s *t* test (H). **Fig. S2, related to Fig. S2.** Mice were divided into six groups: AAV8-NC, NC + CCl_4_, lnc-Helf-sh1#, lnc-Helf-sh1# + CCl_4_, lnc-Helf-sh2#, and lnc-Helf-sh2# + CCl_4_. Mice were injected with AAV8-lnc-Helf-shRNAs or AAV8-NC virus 2 weeks after the first injection of CCl_4_ via tail vein. After CCl_4_ treatment for 8 weeks, (A–D) the volcano map and GO analysis of differentially expressed mRNAs in lnc-Helf-sh1# + CCl_4_ mice and lnc-Helf-sh2# + CCl_4_ mice are shown, compared with NC + CCl_4_ mice. **Fig. S3, related to Fig. S2.** Mice were divided into six groups: AAV8-NC, NC + CCl_4_, lnc-Helf-sh1#, lnc-Helf-sh1# + CCl_4_, lnc-Helf-sh2# and lnc-Helf-sh2# + CCl_4_. Mice were injected with AAV8-lnc-Helf-shRNAs or AAV8-NC virus 2 weeks after the first injection of CCl_4_ via tail vein. After CCl_4_ treatment for 8 weeks (A), IHC for COL1α1 and TGFβ are shown; scale bar is 100 μm for 40× and 400 μm for 10× magnifications . (B) qRT–PCR was used to assess the expression of *Acta2*, *Col1α1*, *Mmp2*, *Timp1*, and *Tgfβ1*. (C, D) Serum ALT and AST was examined. Data are presented as mean ± SEM.^*/#^*p* < 0.05. *p < 0.05 for AAV8-NC. ^#^*p* < 0.05 for NC + CCl_4_, one-way ANOVA (B-D). **Fig. S4, related to Fig. S2.** Mice were divided into six groups: AAV8-NC, NC + CCl_4_, lnc-Helf-sh1#, lnc-Helf-sh1# + CCl_4_, lnc-Helf-sh2#, and lnc-Helf-sh2# + CCl_4_. Mice were injected with AAV8-lnc-Helf-shRNAs or AAV8-NC virus 2 weeks after the first injection of CCl_4_ via tail vein. After CCl_4_ treatment for 8 weeks, (A) western blot was used to determine the expression of CD11b, TNF-α, IL-1β, and MCP1. GAPDH was used as an internal control. (B, C) qRT–PCR was used to assess the RNA level of proinflammatory genes (*Tnf-α*, *Mcp1*, *Il-1β*, and *Il-6*) and proliferation-related genes (*Pcna*, *Ki67*, *Cyclin D1*, and *Bcl-2*) in livers of each group. (D) IHC for CD11b, F4/80, TNF-α, IL-1β, LY6C, and PCNA; scale bar is 100 μm for 40× and 400 μm for 10× magnifications. Data are presented as mean ± SEM. ^*/#^*p* < 0.05. *p < 0.05 for AAV8-NC. ^#^*p* < 0.05 for NC + CCl_4_, one-way ANOVA (B and C). **Fig. S5, related to Fig. S3**. Mice were divided into six groups: AAV8-NC, NC + BDL, lnc-Helf-sh1#, lnc-Helf-sh1# + BDL, lnc-Helf-sh2# and lnc-Helf-sh2# + BDL. Mice were injected with AAV8-lnc-Helf-shRNAs or AAV8-NC virus 2 days before sham operation or bile duct ligate operation via tail vein. After 21 days of operation, (A) qRT–PCR was used to examine the RNA level of *lnc-Helf* in livers of each group. (B, C) Serum AST and ALT was examined. (D) qRT–PCR was used to assess the RNA level of *Pcna*, *Ki67*, *Cyclin D1*, and *Bcl-2* in livers of each group. Data are presented as mean ± SEM. ^*/#^*p* < 0.05. *p < 0.05 for AAV8-NC. ^#^*p* < 0.05 for NC + BDL, one-way ANOVA (A-D). **Fig. S6, related to Fig. S4.** (A) Quantitative analysis of western blot of Fig. S4B. (B) Primary HSCs at day 2 were infected with lenti-control and lnc-Helf-shRNA or lenti-lnc-Helf for 48 h, following the treatment with 10 ng/ml TGF-β1. The expression and location of COL1α1 was assessed by confocal microscopy. Scale bar is 20 μm. (C) Quantitative analysis of western blot of Fig. S4F. (D) Primary HSCs at day 2 were infected with lenti-control and lenti-lnc-Helf for 48 h, following the treatment with 10 ng/ml TGF-β1. The expression and location of COL1α1 was assessed by confocal microscopy. Scale bar is 20 μm. (E–G) LX-2 cells were infected with lenti-control or lenti-lnc-HELF for 48 h, following the treatment with TGF-β1 for 24 h. qRT–PCR was used to assess the expression of *lnc-HELF*, *ACTA2*, *COL1α1*, *COL1α2*, *TIMP1*, *TGF-β1*, *PCNA*, *KI67*, and *CYCLIN D1* (E); western blot was used to determine the expression of the protein level of α-SMA, MMP2, CYCLIN D1, CYCLIN B1, and PCNA (F). GAPDH was used as an internal control. Cell proliferation was detected by CCK8 (G). (H) Primary HSCs at day 3 were infected with lenti-control and lenti-lnc-Helf for 96 h, cell proliferation was detected by CCK8. Data are presented as mean ± SEM. ^*/#^*p* < 0.05. *p < 0.05 for LV-control. ^#^*p* < 0.05 for LV-control + TGF-β1, one-way ANOVA (A, C, and E) and unpaired Student’s *t* test (G and H). **Fig. S7, related to Fig. S5.** (A) Mouse primary HMs transfected with siRNA for 24 h, qRT–PCR analysis was used to detect the RNA level of *Mrc1*, *Arg1*, *Cd163*, and *Il10*. (B) HMs were transfected with pcDNA3.1-lnc-Helf or pcDNA3.1 for 48 h, qRT–PCR was used to assess the expression of *Mrc1*, *Arg1*, *Cd163*, and *Il10*. (C) RAW264.7 cells were transfected with pcDNA3.1 or pcDNA3.1-lnc-Helf for 48 h, qRT–PCR was used to assess the expression of *lnc-Helf*, *Tnf-α*, *Mcp-1*,* Il-1β*, *Il-6*, *Pcna*, *Ki67*, *Cyclin D1*, *Cyclin E1*, *Mrc1*, *Arg1*, and *Il10*. (D) RAW264.7 cells were transfected with pcDNA3.1 or pcDNA3.1-lnc-Helf for 48 h following treatment with 20 ng/ml IFN-γ for 24 h. Mature supernatant IL-1β level was detected by ELISA. Data are presented as mean ± SEM. ^*/#^*p* < 0.05. *p < 0.05 for pcDNA3.1. ^#^*p* < 0.05 for pcDNA3.1 + IFN-γ, unpaired Student’s *t* test (A–C) and one-way ANOVA (D). **Fig. S8, related to Fig. S6.** (A) Mice were divided into six groups: AAV8-NC, NC + BDL, lnc-Helf-sh1#, lnc-Helf-sh1# + BDL, lnc-Helf-sh2#, and lnc-Helf-sh2# + BDL. Western blot was used to determine the protein level of phos-AKT (Thr308), phos-c-Raf1 (Ser259), phos-GSK-3β (Ser9), and AKT in liver tissues of each group. (B) LX-2 cells were infected with lenti-control or lenti-lnc-HELF for 48 h following treatment with 10 ng/ml TGF-β1 for 24 h. Western blot was used to determine the protein level of phos-AKT (Thr308) and AKT. GAPDH was used as an internal control. (C) RAW264.7 cells treated with or without the AKT inhibitor MK2206 were transfected with pcDNA3.1 or pcDNA3.1-lnc-Helf for 72 h. qRT–PCR was used to detect the expression of *lnc-Helf*, *Tnf-α*, *Mcp-1*, *Il-1β*, *Il-6*, *Pcna*, *Cyclin D1*, and *Cyclin E1*. Data are presented as mean ± SEM. ^*/#^*p* < 0.05. *p < 0.05 for pcDNA3.1 + DMSO. ^#^*p* < 0.05 for pcDNA3.1-lnc-Helf + DMSO, one-way ANOVA (C). **Fig. S9, related to Fig. S7.** The expression and location of α-SMA in lnc-Helf-increased HSCs simultaneously transfected with siPTBP1 was assessed by confocal microscopy. Scale bar is 20 μm. **Fig. S10, related to Fig. S8.** (A, B) Prediction of the interaction probabilities between lnc-Helf and PTBP1 using the RPIseq and prediction of lncRNA–protein interactions database. (C) qRT–PCR was used to detect the RNA level of *PTBP1* in livers of healthy people (*n* = 6) and fibrotic patients (*n* = 28). (D, E) The correlation of *PTBP1*, *lnc-HELF*, and *PIK3R5* was assessed by Pearson correlation analysis, *n* = 34. (F–I) Mice were divided into six groups: AAV8-NC, NC + CCl_4_/BDL, lnc-Helf-sh1#, lnc-Helf-sh1# + CCl_4_/BDL, lnc-Helf-sh2#, and lnc-Helf-sh2# + CCl_4_/BDL. qRT–PCR was used to detect the RNA level of *Pik3r5* and *Ptbp1* in livers of each group. Data are presented as mean ± SEM. ^*/#^*p* < 0.05. *p < 0.05 for AAV8-NC. ^#^*p* < 0.05 for NC + CCl_4_/BDL, one-way ANOVA (F–I). **Fig. S11, related to Fig. S8.** (A, B) Primary HSCs at day 2 were infected with lenti-control or lnc-Helf-shRNA or lenti-lnc-Helf for 48 h, following the treatment with TGF-β1 for 24 h. qRT–PCR was used to detect the RNA level of *Pik3r5*. (C) LX-2 cells were infected with lenti-control or lenti-lnc-HELF for 48 h, following the treatment of TGF-β1 for 24 h. qRT–PCR was used to assess the expression of *PIK3R5*. (D) Mouse primary HMs were transfected with lnc-Helf siRNA for 24 h following treatment of 20 ng/ml IFN-γ for 24 h. qRT–PCR was used to assess the expression of *Pik3r5*. (E, F) Mouse primary HMs and RAW264.7 cells were transfected with pcDNA3.1 or pcDNA3.1-lnc-Helf for 48 h, qRT–PCR was used to assess the expression of *Pik3r5*. (G) qRT–PCR was used to assess the expression of *Pik3r5* in lnc-Helf-overexpressed RAW264.7 cells simultaneously transfected with siPTBP1. Data are presented as mean ± SEM. ^*/#^*p* < 0.05. *p < 0.05 for LV-control or si-NC or pcDNA3.1or pcDNA3.1 + si-NC. ^#^*p* < 0.05 for LV-control + TGF-β1/IFN-γ or pcDNA3.1-lnc-Helf + si-NC, one-way ANOVA (A–D, and G) and unpaired Student’s *t* test (E and F). **Fig. S12.** Schematic diagram shows the function and mechanism of lnc-Helf in the progression of hepatic inflammation and fibrosis. Upon liver injury, increased lnc-Helf binds with PTBP1 to promote its interaction with PIK3R5 mRNA, resulting in increased stability and activating the AKT pathway, thus promotes HSCs and HMs activation and proliferation, which augments hepatic inflammation and fibrosis. **Table S1**. Clinical characteristics of patients. **Table S2**. The results of mass spectrometry from proteins pulled down by the sense and antisense of lnc-Helf. **Table S3**. Cloning primers for lnc-Helf. **Table S4** siRNA sequences. **Table S5** qRT–PCR primers. **Table S6** RACE primers for lnc-Helf.

**Additional file 2:** The results of mass spectrometry from proteins pulled down by the sense and antisense of lnc-Helf.

## Data Availability

All the data used and analyzed during this study are available from the corresponding author upon reasonable request.

## Abbreviations

LncRNA: Long noncoding RNA
CCl_4_: Carbon tetrachloride
BDL: Bile duct ligation
HC: Hepatocyte
HSC: Hepatic stellate cell
LSEC: Liver sinusoidal endothelial cell
HM: Hepatic macrophages
KC: Kupffer cell
PTBP1: Polypyrimidine tract-binding protein 1
ECM: Extracellular matrix
RACE: Rapid amplification of cDNA ends
