# Supplementary material for: lncRNA Helf promotes hepatic inflammation and fibrosis by interacting with PTBP1 to facilitate PIK3R5 mRNA stabilization
Source: Cell Mol Biol Lett. 2023 Oct 7;28:77. doi: 10.1186/s11658-023-00492-3 (PMC10560431; doi:10.1186/s11658-023-00492-3)
Supplement: Supplementary file 1 — Additional file 1: Fig. S1, related to Fig. S1. (A) The fold change of lnc-Helf was shown according to the microarray data. (B, C) The nucleotide sequence of mouse lnc-Helf and human lnc-HELF. (D) The comparison between human and mouse lnc-Helf sequences. (E) The nuclei or cytoplasm of primary HSCs was isolated, and qRT–PCR analysis detected the expression of lnc-Helf, Neat1, and Gapdh. (F) The intracellular localization of lnc-Helf in primary HSCs was measured by RNA-FISH assays; scale bar is 10 μm. (G) qRT–PCR analysis of lnc-Helf in AML12 cells, LX-2 cells, RAW264.7 cells, BMMs, and HUVECs (H) qRT–PCR was used to assess the expression of lnc-Helf and Acta2 in primary HSCs cultured at day 2 treated with 10 ng/ml TGF-β for 24 h. (I–L) The correlations of lnc-HELF, ACTA2, COL1α1, ALT, and AST were assessed by Pearson correlation analysis, n = 34. *p < 0.05, unpaired Student’s t test (H). Fig. S2, related to Fig. S2. Mice were divided into six groups: AAV8-NC, NC + CCl4, lnc-Helf-sh1#, lnc-Helf-sh1# + CCl4, lnc-Helf-sh2#, and lnc-Helf-sh2# + CCl4. Mice were injected with AAV8-lnc-Helf-shRNAs or AAV8-NC virus 2 weeks after the first injection of CCl4 via tail vein. After CCl4 treatment for 8 weeks, (A–D) the volcano map and GO analysis of differentially expressed mRNAs in lnc-Helf-sh1# + CCl4 mice and lnc-Helf-sh2# + CCl4 mice are shown, compared with NC + CCl4 mice. Fig. S3, related to Fig. S2. Mice were divided into six groups: AAV8-NC, NC + CCl4, lnc-Helf-sh1#, lnc-Helf-sh1# + CCl4, lnc-Helf-sh2# and lnc-Helf-sh2# + CCl4. Mice were injected with AAV8-lnc-Helf-shRNAs or AAV8-NC virus 2 weeks after the first injection of CCl4 via tail vein. After CCl4 treatment for 8 weeks (A), IHC for COL1α1 and TGFβ are shown; scale bar is 100 μm for 40× and 400 μm for 10× magnifications . (B) qRT–PCR was used to assess the expression of Acta2, Col1α1, Mmp2, Timp1, and Tgfβ1. (C, D) Serum ALT and AST was examined. Data are presented as mean ± SEM.*/#p < 0.05. *p < 0.05 for AAV8-NC. [file 11658_2023_492_MOESM1_ESM.docx]

**Supplementary Information**

**Supplementary materials** **and methods**

**Cell culture**

The murine immortalized macrophages RAW264.7 cells, human hepatic stellate cell line LX-2, HUVECs, AAV293 and HEK293T cells were cultured in DMEM supplemented with 10% fetal bovine serum (FBS), penicillin (100 U/ml) and streptomycin (100 μg/ml). BMMs, which were isolated from the femur, were cultured in α-MEM (Gibco, Gaithersburg, MD, USA) containing 10% FBS, penicillin (100 U/ml) and streptomycin (100 μg/ml) and 10 ng/mL murine macrophage colony stimulating factor (M-CSF) (PeproTech, Rocky Hill, USA) for 6 days. Medium was changed every 2 days. The non-tumorigenic mouse hepatocyte cell line AML12 was maintained in DMEM/F-12 (Gibco, Gaithersburg, MD, USA) supplemented with 10% FBS, 1×insulin-transferrin-sodium selenite media supplement (ITS; Sigma-Aldrich), dexamethasone (40ng/ml), penicillin (100U/ml) and streptomycin (100μg/ml). All cells were cultured at 37 °C in an atmosphere containing 5% CO_2_.

**Plasmid construction**

The full-length lnc-Helf cDNA were sequentially amplified by PCR and ligated into the lentiviral shuttle pCCL.PPT.hPGK.IRES.eGFP/pre or pcDNA3.1(+) to generate the over-expression plasmid (Lv-lnc-Helf or pcDNA3.1-lnc-Helf). The empty plasmid was used as control. Oligos encoding shRNAs specific for lnc-Helf and the negative control shRNA were ligated into the lentiviral shuttle pCCL.PPT.hPGK.GFP.Wpre (lenti-lnc-Helf-shRNA and lenti-Ctrl) or p-AAV-sh[control] (addgene, #75438). These plasmids were used to produce lentivirus in HEK-293T cells with the packaging plasmids pMD2.BSBG, pMDLg/pRRE and pRSV-REV. AAV8 virus were produced by transfecting AAV-293 cells using polyethylenimine (PEI) with an AAV vector plasmid and helper plasmids including pAAV2/8 (addgene, #112864), pAdDeltaF6 (addgene, #112867). The primers used are shown in Supplementary Table 3.

**Histology and Immunohistochemistry (IHC)**

Livers were harvested, fixed in 10% neutral buffered formalin for 48 h, dehydrated, and embedded in paraffin. Sections (5 μm) were stained with hematoxylin and eosin (H&E), Sirius red and Masson staining. According to the above results, three sections were chosen from each group for IHC analysis. Briefly, dewaxed sections were incubated in antigen retrieval buffer (pH 6.0) for 5 min at 108 °C and submitted to 3% H_2_O_2_ solution for 15 min. Following this, sections were then treated with normal goat serum for 20 min to block the nonspecific immunoreactivity and subjected to incubation with primary antibodies α-SMA (1:50, rabbit polyclonal, Abcam, ab5694), collagen1 (1:1000, rabbit polyclonal, Abcam, ab34710), TGFβ (1:50, rabbitpolyclonal, Abcam, ab66043), CD11b (1:100, rabbit monoclonal, Abcam, ab133357), F4/80 (1:50, rat monoclonal, Abcam, ab16911), LY6C (1:200, rat monoclonal, Novus, NBP2-00441), TNF-α (1:50, mouse monoclonal, Santa Cruz, sc-52746), IL-1β (1:100, mouse monoclonal, Cell Signaling Technology, #12242) or PCNA (1:1000, rabbit monoclonal, Cell Signaling Technology, #13110) overnight at 4 °C. In addition, sections were incubated with secondary antibody (1:500) (horseradish peroxidase-conjugated anti-rabbit IgG) and the reaction products were visualized using diaminobenzidine (DAB) and monitored by microscopy.

**Hydroxyproline assay**

Hydroxyproline content in liver tissue was measured by hydroxyproline assay kit purchased from Nan Jing Jian Cheng Biochemical Institute (Nanjing, China) according to the manufacturer’s instructions.

**IL-1β and liver enzyme measurement**

The IL-1β level in cell culture media and the alanine aminotransferase (ALT) and aspartate aminotransferase (AST) levels in the serum were assessed using commercial assay kits (Nanjing Jiancheng Corp., Nanjing, China) according to the manufacturer’s protocols.

**Cell transfection and ActD treatment**

For gene knockdown analysis, cells at 30-50% confluency were transfected with 20 nM of siRNAs by using lipofectamine MAX according to the manufacturer’s instructions (Invitrogen). siRNAs targeting the lnc-Helf and PTBP1 sequences and non-targeting siRNA were obtained from GenePharma Biological Technology (Shanghai, China). For gene overexpression analysis, pcDNA3.1-Helf and negative control vectors were transfected into cells using Lipofectamine 2000 according to the manufacturer’s instructions (Invitrogen). Target sequences of these siRNA are listed in Supplementary Table 4. The RAW 264.7 cells seeded in 12-well plates were transfected with si-PTBP1 or si-NC with or without pcDNA3.1-lnc-Helf or control pcDNA3.1 using lipofectamine MAX. After culturing for 48 h, cells were treated with 1 μg/ml actinomycin D (ActD) for 0 h, 2 h and 4 h respectively.

**CCK8 Assay**

Cell viability was assessed using CCK-8 kit according to the manufacturer’s protocol. Briefly, LX-2, RAW 264.7 or primary HSCs were plated in a clear bottom 96-well plate with six multiple pores. After culturing overnight, cells were transfected with pcDNA3.1-lnc-Helf and pcDNA3.1 or lentivirus over-expressing lnc-HELF and control lentivirus for 48 h, 72 h and 96 h respectively. At a specific time point, add 10 µl CCK-8 solution to each well and incubate for 2 h at 37ºC, 5% CO_2_ incubator. The absorbance was measured at 450 nm.

**Quantitative real-time polymerase chain reaction**

Total RNA extracted from liver tissues or cells with Trizol reagent (Takara, Dalian, China) and treated with DNaseI (Invitrogen). All RNAs were reverse-transcribed into cDNA using AMV Reverse Transcriptase (Thermo Fisher Scientific, Basingstoke, UK). For real-time PCR, all reactions were performed with SYBR Green master mix (Takara, Dalian, China) according to the manufacturer’s instructions in Light Cycler®96 Real-Time PCR System (Roche). The expression level of each gene was normalized to internal control GAPDH. And the specific primers for real-time PCR are provided in Supplementary Table 5.

**Western blot**

The tissues and cells lysates were obtained through RIPA buffer lysis supplemented with protease inhibitor cocktail, 1% phenylmethanesulfonyl fluoride (PMSF) and 1% phosphatase inhibitor. Protein concentrations were measured by the BCA^TM^ Protein Assay Kit (Bio-Rad Laboratories, Hercules, CA, USA) using BSA as standard. Next, the total proteins were immunoblotted with the appropriate antibodies. The antibodies used in this study were: MMP2 (rabbit monoclonal, Abcam, ab92536, 1:2000), α-SMA (rabbit polyclonal, Abcam, ab5694, 1:1000), TIMP1 (mouse monoclonal, Santa Cruz, sc-21734; 1:1000), GAPDH (mouse monoclonal, Abcam, ab8245; 1:8000), CD11b (rabbit monoclonal, Abcam, ab133357; 1:1000), TNF-α (mouse monoclonal, Santa Cruz, sc-52746; 1:1000), IL-1β (mouse monoclonal, Cell Signaling Technology, #12242; 1:1000), MCP1 (rabbit polyclonal, Cell Signaling Technology, #2029; 1:1000), Cyclin D1 (rabbit monoclonal, Abcam, ab134175; 1:5000), Cyclin B1 (rabbit monoclonal, Abcam, ab181593; 1:1000), PCNA (rabbit monoclonal, Cell Signaling Technology, #13110; 1:1000), phos-AKT (Thr308) (rabbit monoclonal, Cell Signaling Technology, #13038; 1:1000), AKT (rabbit monoclonal, Cell Signaling Technology, #4691; 1:1000), phos-c-Raf (Ser259) (rabbit polyclonal, Cell Signaling Technology, #9421; 1:1000), phos-GSK-3β(Ser9) (rabbit monoclonal, Cell Signaling Technology, #5558; 1:1000) and PTBP1 (Goat polyclonal, Abcam, ab5642; 1:2000). Specific proteins were visualized using an enhanced chemiluminescence (ECL, Merck Millipore, Darmstadt, Germany) western blot detection system (Millipore). GAPDH was used as an internal control.

**Confocal microscopy**

Freshly isolated primary HSCs and HMs cells were plated on poly-lysine-pre-coated glass cover slips and incubated overnight at 37°C to reach typical adhesion and spreading. The HMs cells were transfected with siRNA targeting lnc-Helf or siRNA-control for 24 h, recombinant treating with 20 ng/ml IFN-γ (PeproTech) for additional 24 h. The HSCs cells were transfected with lentivirus over-expressing lnc-Helf, knowdown lnc-Helf or control lentivirus for 48 h, recombinant treating with 10 ng/ml TGFβ (PeproTech) for additional 24 h. After washing with PBS, cells were fixed in 4% paraformaldehyde for 15 min, permeabilized in 1% Triton X-100 and incubated with normal goat serum for 20 min to block the nonspecific immunoreactivity at room temperature. Then the cells were incubated with primary antibody α-SMA (1:200, rabbit polyclonal, Abcam, ab5694), collagen1 (1:500, rabbit polyclonal, Abcam, ab34710) and TNF-α (1:50, mouse monoclonal, Abcam, ab1793) overnight at 4 °C. Alexa Fluor 594-conjugated secondary antibody in PBS was added and incubated for 1 h in the dark at room temperature. Then cells were washed three times and subjected to incubation with medium containing DAPI (10 μg/ml, Sigma) for 10 min at room temperature followed by washing with PBS. Finally, the cover slips were mounted with an anti-fade mounting medium (P0126, Beyotime, Shanghai, China). All immunofluorescence was then visualized by a confocal microscope (LSM 700).

**Nuclear-cytoplasmic fractionation**

Cytoplasmic and nuclear RNA isolation were performed with PARIS™ Kit (Invitrogen, Grand Island, NY, USA) following the manufacturer’s instruction.

**Fluorescence in situ hybridization (FISH)**

FISH was performed using a FISH Kit (GenePharma) according to the manufacturer’s instructions. Nuclei were stained with DAPI. Images were acquired on a Zeiss confocal microscope LSM700. The sequences of FISH probe were as follows: probe1: 5’-GATCTCAGCGTGACCTATTC-3’, probe 2: 5’-GAGATGCCAGCCTGAGTTCA-3’, probe 3: 5’-CAGGTTGGCACAGCATTGAC-3’, probe 4:5’-AACTCGGGCTGTTTTGCTTT-3’, probe 5: 5’-ATTACCTAGCTGGCTTGTCG-3’, probe 6: 5’-GCAAACATGGCTTAATTTTC-3’.

**5' and 3' rapid amplification of cDNA ends (RACE)**

5'-RACE and 3'-RACE analyses were used to determine the transcriptional initiation and termination sites of lnc-Helf according to the manufacturer’s protocol of SMARTer™ RACE cDNA Amplification Kit (Clontech, Palo Alto, CA). In brief, RNA isolated from 10-week-old male Balb/c mice was reversed into cDNA for 3’- and 5’-RACE by SMART Scribe Reverse Transcriptase. After PCR amplification of the target fragment, the obtained band was gel purified and cloned into a lineareized pRACE vector using standard techniques. Next, the insert size was checked by restriction digest and subjected to sequencing. The gene-specific primers used for the PCR of the RACE analysis are provided in Supplementary Table 6.

**Supplementary Figures**

**
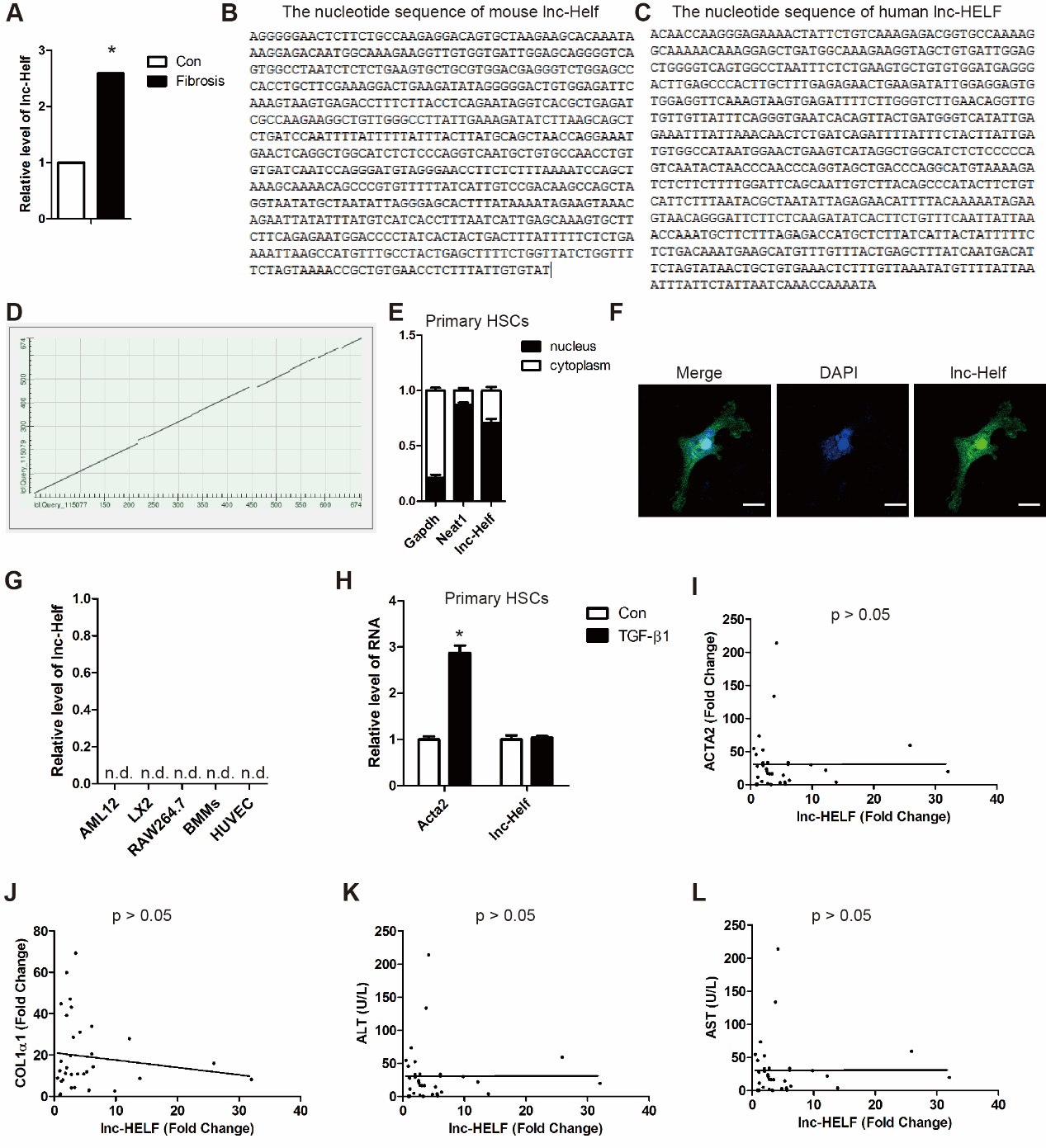
**

**Supplementary Figure 1, related to Figure 1.** (A) The fold change of *lnc-Helf* was showed according to the microarray data. (B, C) The nucleotide sequence of mouse *lnc-Helf* and human *lnc-HELF*. (D) The comparison between human and mouse *lnc-Helf* sequences. (E) The nuclei or cytoplasm of primary HSCs was isolated, and qRT-PCR analysis detected the expression of *lnc-Helf*, *Neat1* and *Gapdh*. (F) The intracellular localization of *lnc-Helf* in primary HSCs was measured by RNA-FISH assays, scale bar, 10 μm. (G) qRT-PCR analysis of *lnc-Helf* in AML12, LX-2, RAW264.7, BMMs and HUVECs cells. (H) qRT-PCR was used to assess the expression of *lnc-Helf* and *Acta2* in primary HSCs cultured at day 2 treated with 10 ng/ml TGF-β for 24 h. (I-L) The correlations of lnc-HELF, ACTA2, COL1α1, ALT and AST were assessed by Pearson correlation analysis, n = 34. **p* < 0.05, Unpaired Student’s t test (H).

**
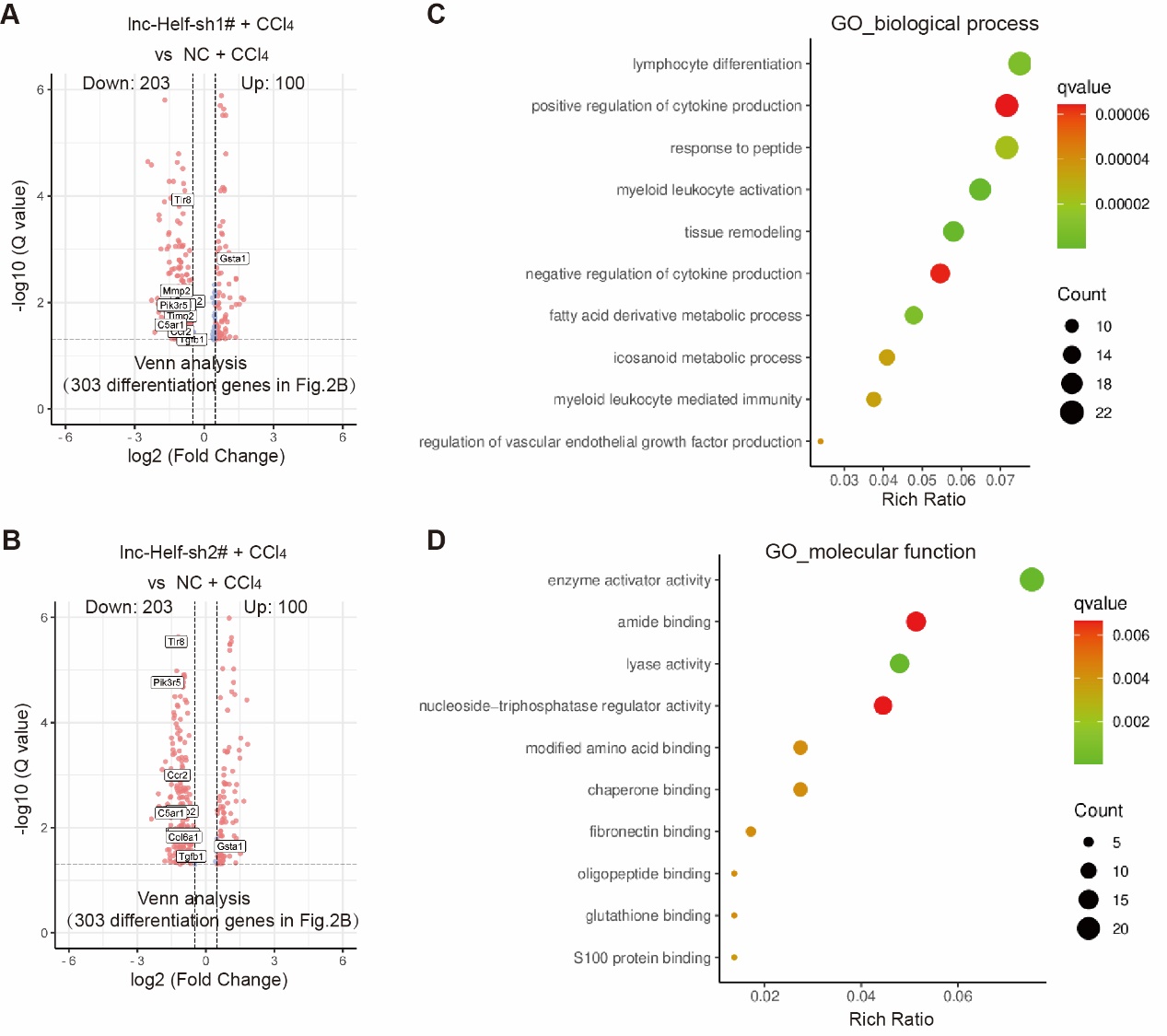
**

**Supplementary Figure 2, related to Figure 2.** Mice were divided into 6 groups: AAV8-NC, NC + CCl_4_, lnc-Helf-sh1#, lnc-Helf-sh1# + CCl_4_, lnc-Helf-sh2# and lnc-Helf-sh2# + CCl_4_. Mice were injected with AAV8-lnc-Helf-shRNAs or AAV8-NC virus 2 weeks after the first injection of CCl_4_ via tail vein. After CCl_4_ treatment for 8 weeks, (A-D) The volcano map and GO analysis of differentially expressed mRNAs in lnc-Helf-sh1# + CCl_4_ mice and lnc-Helf-sh2# + CCl_4_ mice, compared to NC + CCl_4_ mice.


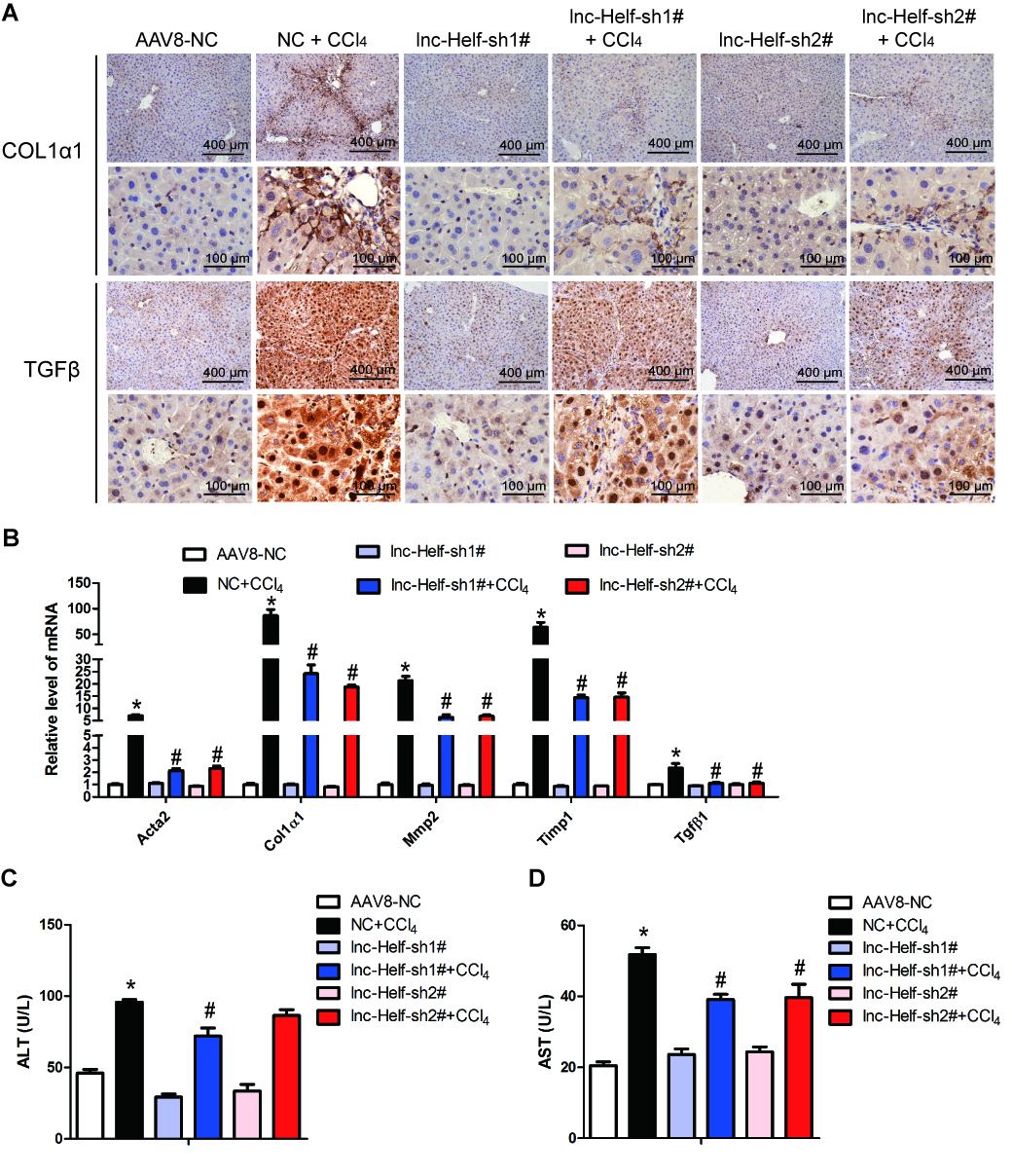


**Supplementary Figure 3, related to Figure 2.** Mice were divided into 6 groups: AAV8-NC, NC + CCl_4_, lnc-Helf-sh1#, lnc-Helf-sh1# + CCl_4_, lnc-Helf-sh2# and lnc-Helf-sh2# + CCl_4_. Mice were injected with AAV8-lnc-Helf-shRNAs or AAV8-NC virus 2 weeks after the first injection of CCl_4_ via tail vein. After CCl_4_ treatment for 8 weeks, (A) IHC for COL1α1 and TGFβ; Scale bar, 100 μm for 40× and 400 μm for 10×. (B) qRT-PCR was used to assess the expression of *Acta2, Col1α1, Mmp2, Timp1* and *Tgfβ1*. (C, D) Serum ALT and AST was examined. Data are presented as mean ± SEM.^*/#^*p* < 0.05. **p*<0.05 for vs AAV8-NC. ^#^*p*<0.05 for vs NC + CCl_4_, one-way ANOVA (B-D).


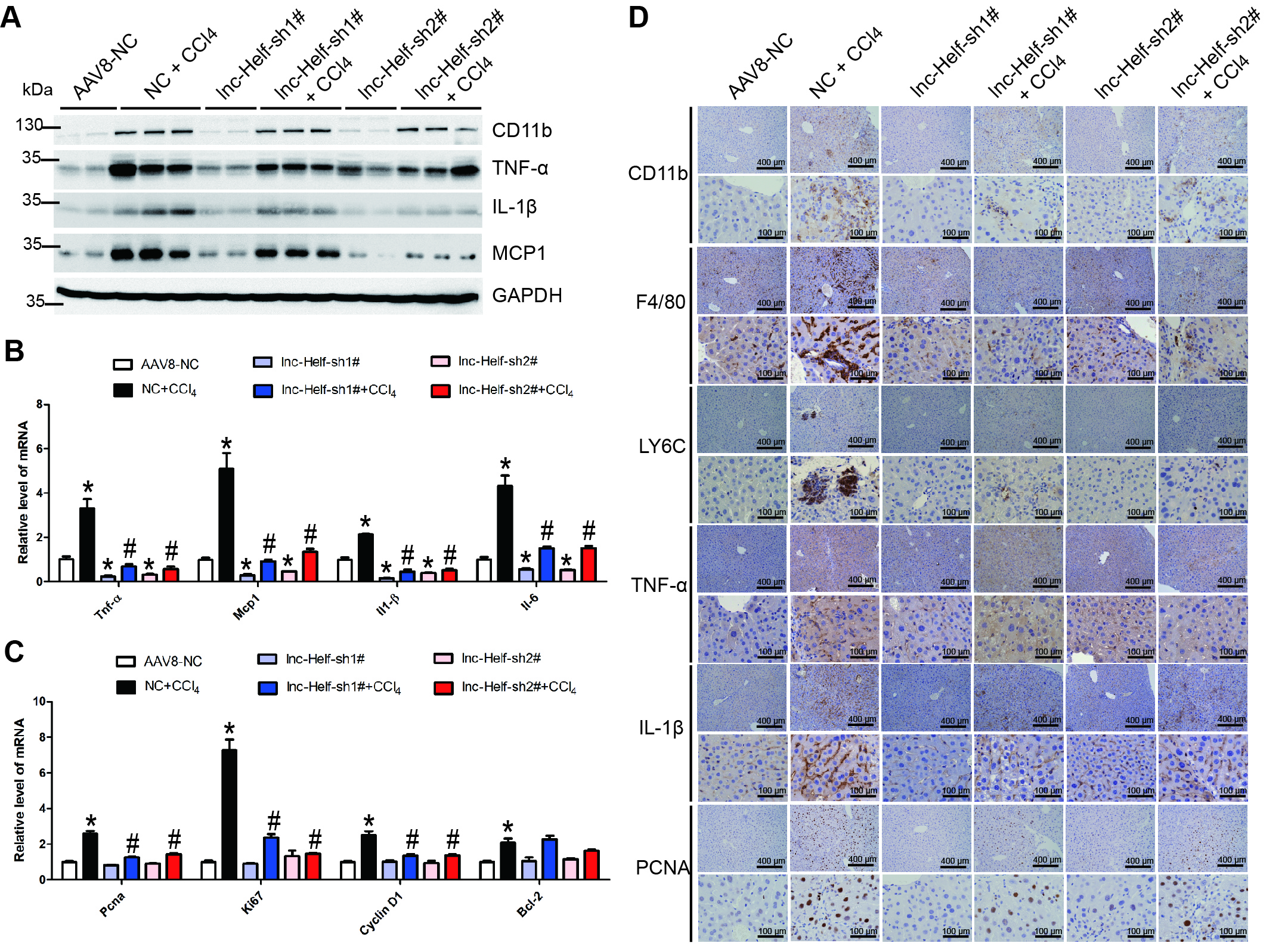


**Supplementary Figure 4, related to Figure 2.** Mice were divided into 6 groups: AAV8-NC, NC + CCl_4_, lnc-Helf-sh1#, lnc-Helf-sh1# + CCl_4_, lnc-Helf-sh2# and lnc-Helf-sh2# + CCl_4_. Mice were injected with AAV8-lnc-Helf-shRNAs or AAV8-NC virus 2 weeks after the first injection of CCl_4_ via tail vein. After CCl_4_ treatment for 8 weeks, (A) Western blot was used to determine the expression of CD11b, TNF-α, IL-1β and MCP1. GAPDH was used as an internal control. (B, C) qRT-PCR was used to assess the RNA level of pro-inflammatory genes (*Tnf-α, Mcp1, Il-1β* and *Il-6*) and proliferation-related genes (*Pcna, Ki67, Cyclin D1* and *Bcl-2*) in livers of each group. (D) IHC for CD11b, F4/80, TNF-α, IL-1β, LY6C and PCNA; Scale bar, 100 μm for 40× and 400 μm for 10×. Data are presented as mean ± SEM. ^*/#^*p* < 0.05. **p*<0.05 for vs AAV8-NC. ^#^*p*<0.05 for vs NC + CCl_4_, one-way ANOVA (B and C).

**
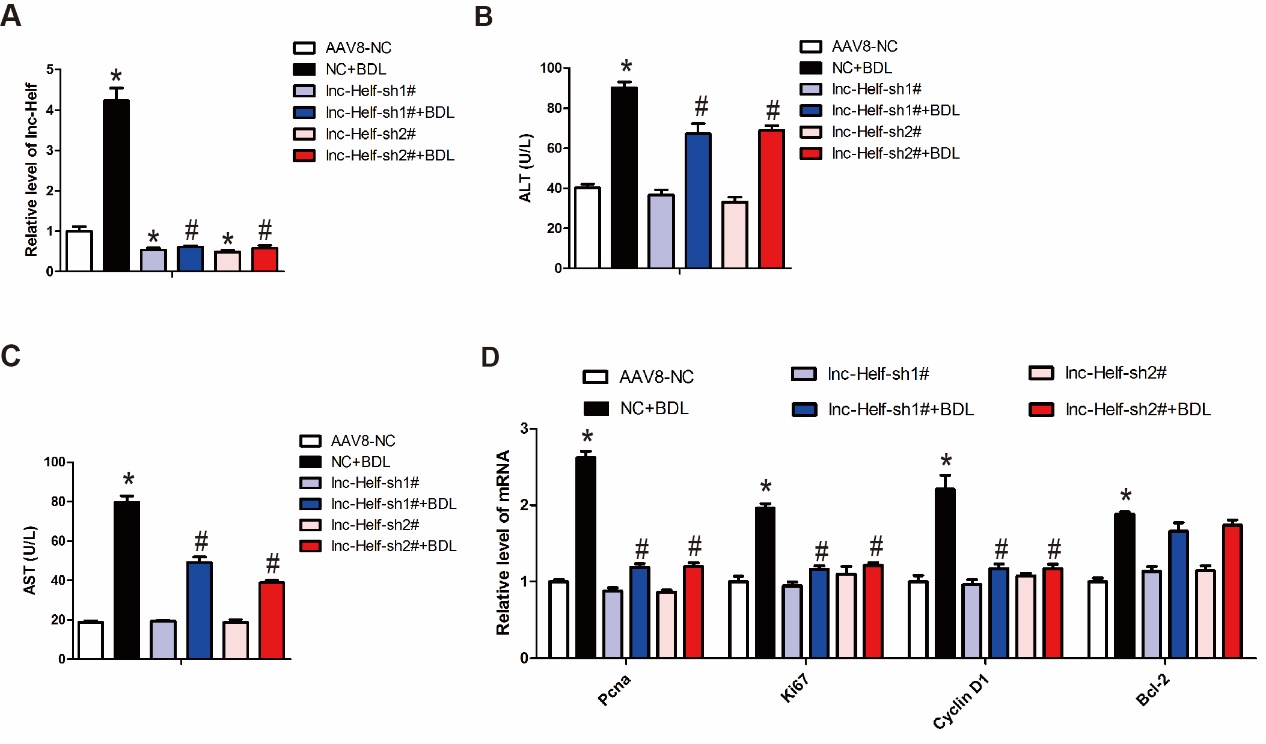
Supplementary Figure 5, related to Figure 3.** Mice were divided into 6 groups: AAV8-NC, NC + BDL, lnc-Helf-sh1#, lnc-Helf-sh1# + BDL, lnc-Helf-sh2# and lnc-Helf-sh2# + BDL. Mice were injected with AAV8-lnc-Helf-shRNAs or AAV8-NC virus 2 days before sham operation or bile duct ligate operation via tail vein. After 21 days of operation, (A) qRT-PCR was used to examine the RNA level of *lnc-Helf* in livers of each group. (B, C) Serum AST and ALT was examined. (D) qRT-PCR was used to assess the RNA level of *Pcna, Ki67, Cyclin D1* and *Bcl-2* in livers of each group. Data are presented as mean ± SEM. ^*/#^*p* < 0.05. **p*<0.05 for vs AAV8-NC. ^#^*p*<0.05 for vs NC + BDL, one-way ANOVA (A-D).

**
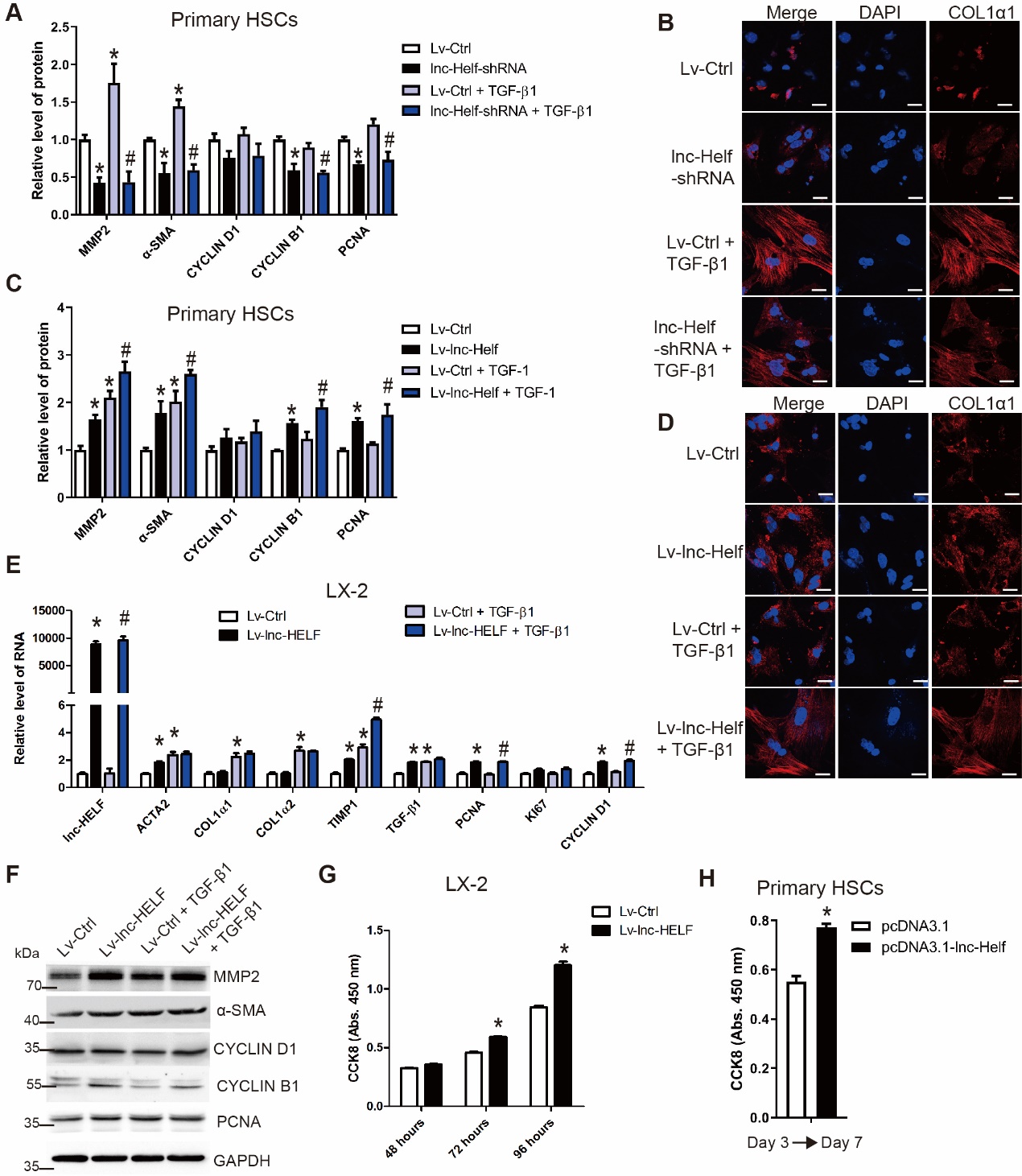
**

**Supplementary Figure 6, related to Figure 4.** (A) Quantitative analysis of Western blot of Figure 4B. (B) Primary HSCs at day 2 were infected with lenti-Ctrl and lnc-Helf-shRNA or lenti-lnc-Helf for 48 hours, following by the treatment of 10 ng/ml TGF-β1. The expression and location of COL1α1 was assessed by confocal microscopy. Scale bar, 20μm. (C) Quantitative analysis of Western blot of Figure 4F. (D) Primary HSCs at day 2 were infected with lenti-Ctrl and lenti-lnc-Helf for 48 hours, following by the treatment of 10 ng/ml TGF-β1. The expression and location of COL1α1 was assessed by confocal microscopy. Scale bar, 20μm.

(E-G) LX-2 cells were infected with lenti-Ctrl or lenti-lnc-HELF for 48 hours, following by the treatment of TGF-β1 for 24 hours. qRT-PCR was used to assess the expression of *lnc-HELF, ACTA2, COL1α1, COL1α2, TIMP1, TGF-β1, PCNA, KI67* and *CYCLIN D1* (E); Western blot was used to determine the expression of the protein level of α-SMA, MMP2, CYCLIN D1, CYCLIN B1 and PCNA (F). GAPDH was used as an internal control. Cell proliferation was detected by CCK8 (G). (H) Primary HSCs at day 3 were infected with lenti-Ctrl and lenti-lnc-Helf for 96 hours, cell proliferation was detected by CCK8. Data are presented as mean ± SEM. ^*/#^*p* < 0.05. **p*<0.05 for vs Lv-Ctrl. ^#^*p*<0.05 for vs Lv-Ctrl + TGF-β1, one-way ANOVA (A, C, and E) and unpaired Student’s t test (G and H).

**
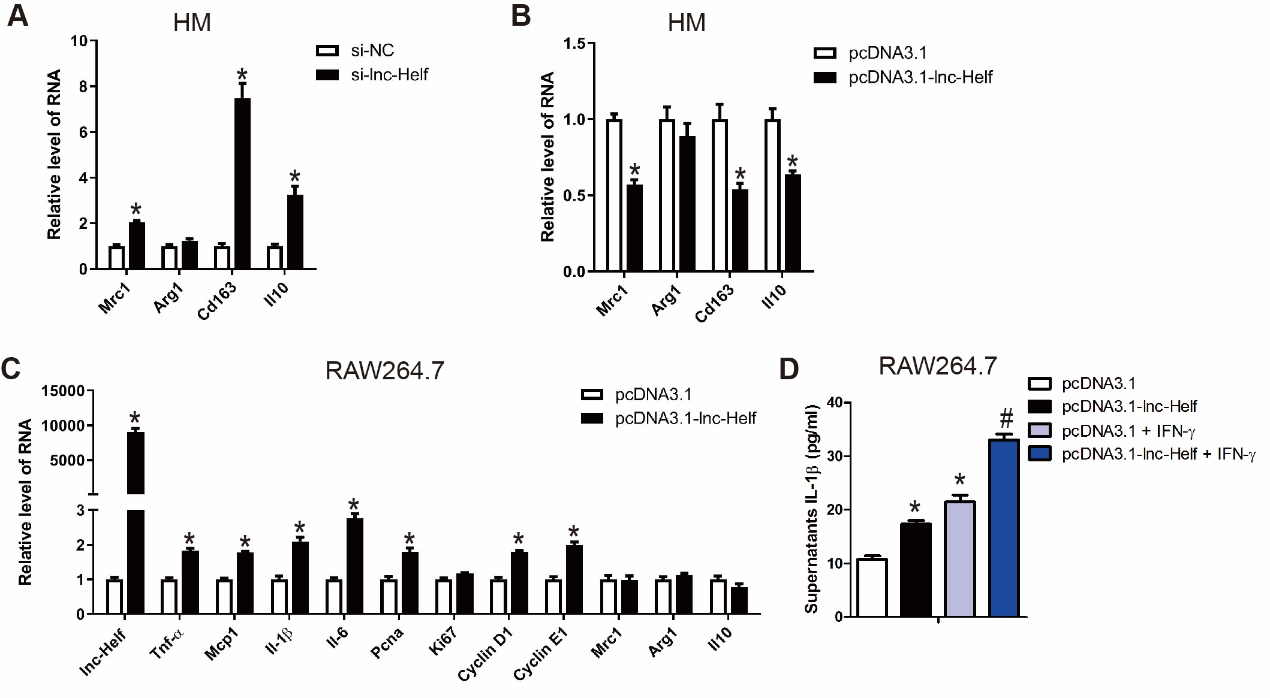
**

**Supplementary Figure 7, related to Figure 5.** (A) Mouse primary HMs transfected with siRNA for 24 hours, qRT-PCR analysis was used to detect the RNA level of *Mrc1, Arg1, Cd163* and *Il10*. (B) HMs were transfected with pcDNA3.1-lnc-Helf or pcDNA3.1 for 48 hours, qRT-PCR was used to assess the expression of *Mrc1, Arg1, Cd163* and *Il10*. (C) RAW264.7 cells were transfected with pcDNA3.1 or pcDNA3.1-lnc-Helf for 48 hours, qRT-PCR was used to assess the expression of *lnc-Helf, Tnf-α, Mcp-1, Il-1β, Il-6, Pcna, Ki67, Cyclin D1*, *Cyclin E1, Mrc1, Arg1* and *Il10*. (D) RAW264.7 cells were transfected with pcDNA3.1 or pcDNA3.1-lnc-Helf for 48 hours following by treated with 20ng/ml IFN-γ for 24 hours. Supernatant mature IL-1β level was detected by ELISA. Data are presented as mean ± SEM. ^*/#^*p* < 0.05. **p*<0.05 for vs pcDNA3.1. ^#^*p*<0.05 for vs pcDNA3.1 + IFN-γ, unpaired Student’s t test (A-C) and one-way ANOVA (D).

**
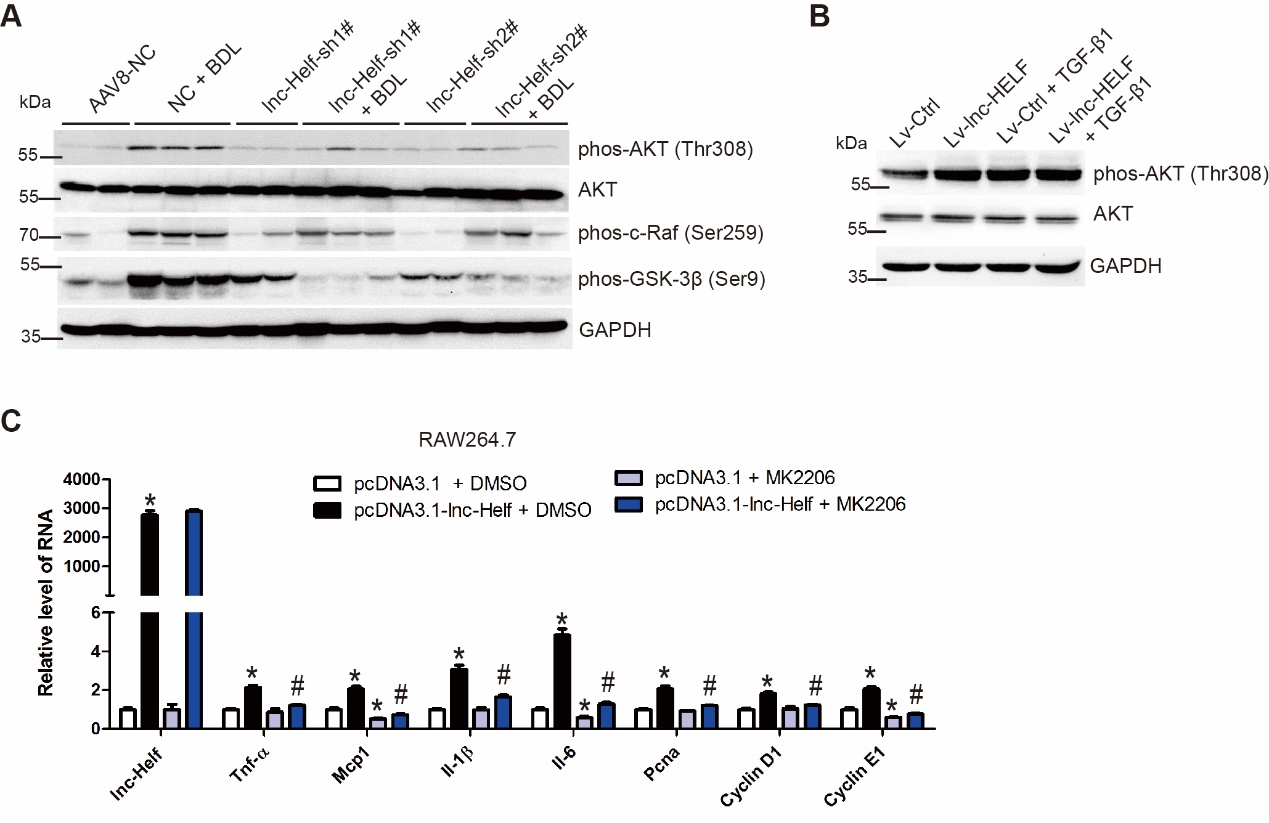
Supplementary Figure 8, related to Figure 6.** (A) Mice were divided into 6 groups: AAV8-NC, NC + BDL, lnc-Helf-sh1#, lnc-Helf-sh1# + BDL, lnc-Helf-sh2# and lnc-Helf-sh2# + BDL. Western blot was used to determine the protein level of phos-AKT (Thr308), phos-c-Raf1 (Ser259), phos-GSK-3β (Ser9) and AKT in liver tissues of each group. (B) LX-2 cells were infected with lenti-Ctrl or lenti-lnc-HELF for 48 hours following by treatment of 10 ng/ml TGF-β1 for 24 hours. Western blot was used to determine the protein level of phos-AKT (Thr308) and AKT. GAPDH was used as an internal control. (C) RAW264.7 cells treated with or without the AKT inhibitor MK2206 were transfected with pcDNA3.1 or pcDNA3.1-lnc-Helf for 72 hours. qRT-PCR was used to detect the expression of *lnc-Helf, Tnf-α, Mcp-1, Il-1β, Il-6, Pcna, Cyclin D1* and *Cyclin E1*. Data are presented as mean ± SEM. ^*/#^*p* < 0.05. **p*<0.05 for vs pcDNA3.1 + DMSO. ^#^*p*<0.05 for vs pcDNA3.1-lnc-Helf + DMSO, one-way ANOVA (C).


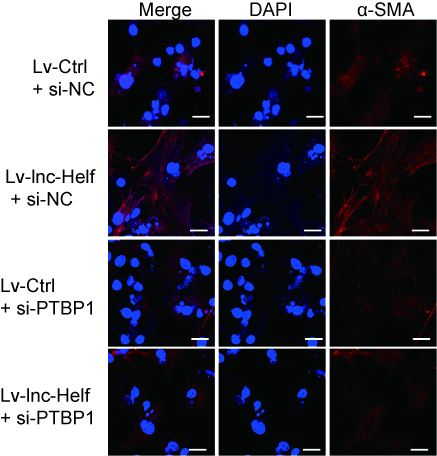


**Supplementary Figure 9, related to Figure 7.** The expression and location of α-SMA in lnc-Helf-increased HSCs simultaneously transfected with siPTBP1 was assessed by confocal microscopy. Scale bar, 20μm.

**
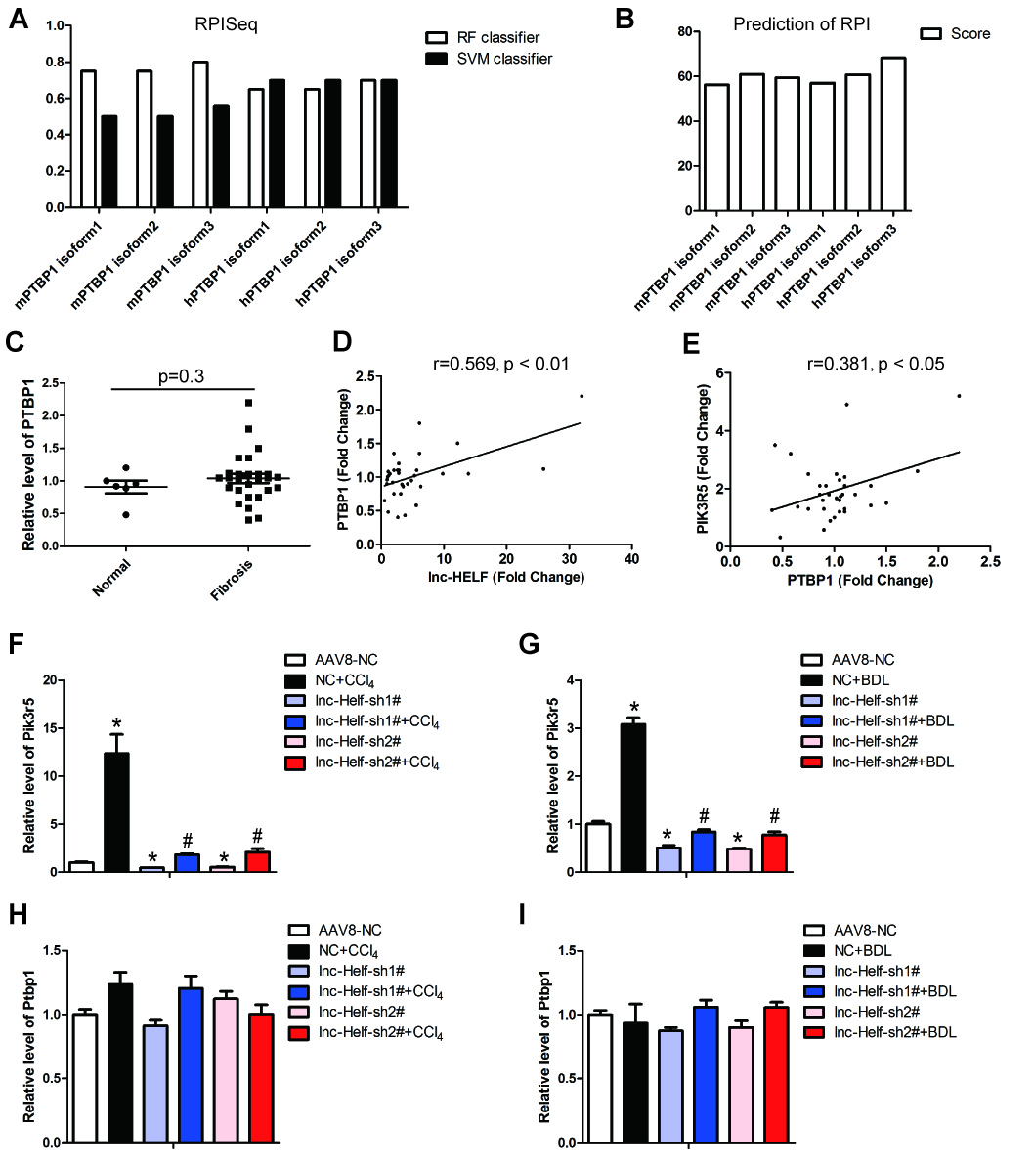
**

**Supplementary Figure 10, related to Figure 8.** (A, B) Prediction of the interaction probabilities between lnc-Helf and PTBP1 using the RPISeq and Prediction of lncRNA-protein interactions database. (C) qRT-PCR was used to detect the RNA level of *PTBP1* in livers of healthy people (n = 6) and fibrotic patients (n = 28). (D, E) The correlation of *PTBP1*, *lnc-HELF* and *PIK3R5* was assessed by Pearson correlation analysis, n = 34. (F-I) Mice were divided into 6 groups: AAV8-NC, NC + CCl_4_/BDL, lnc-Helf-sh1#, lnc-Helf-sh1# + CCl_4_/BDL, lnc-Helf-sh2# and lnc-Helf-sh2# + CCl_4_/BDL. qRT-PCR was used to detect the RNA level of *Pik3r5* and *Ptbp1* in livers of each group. Data are presented as mean ± SEM. ^*/#^*p* < 0.05. **p*<0.05 for vs AAV8-NC. ^#^*p*<0.05 for vs NC + CCl_4_/BDL, one-way ANOVA (F-I).

**
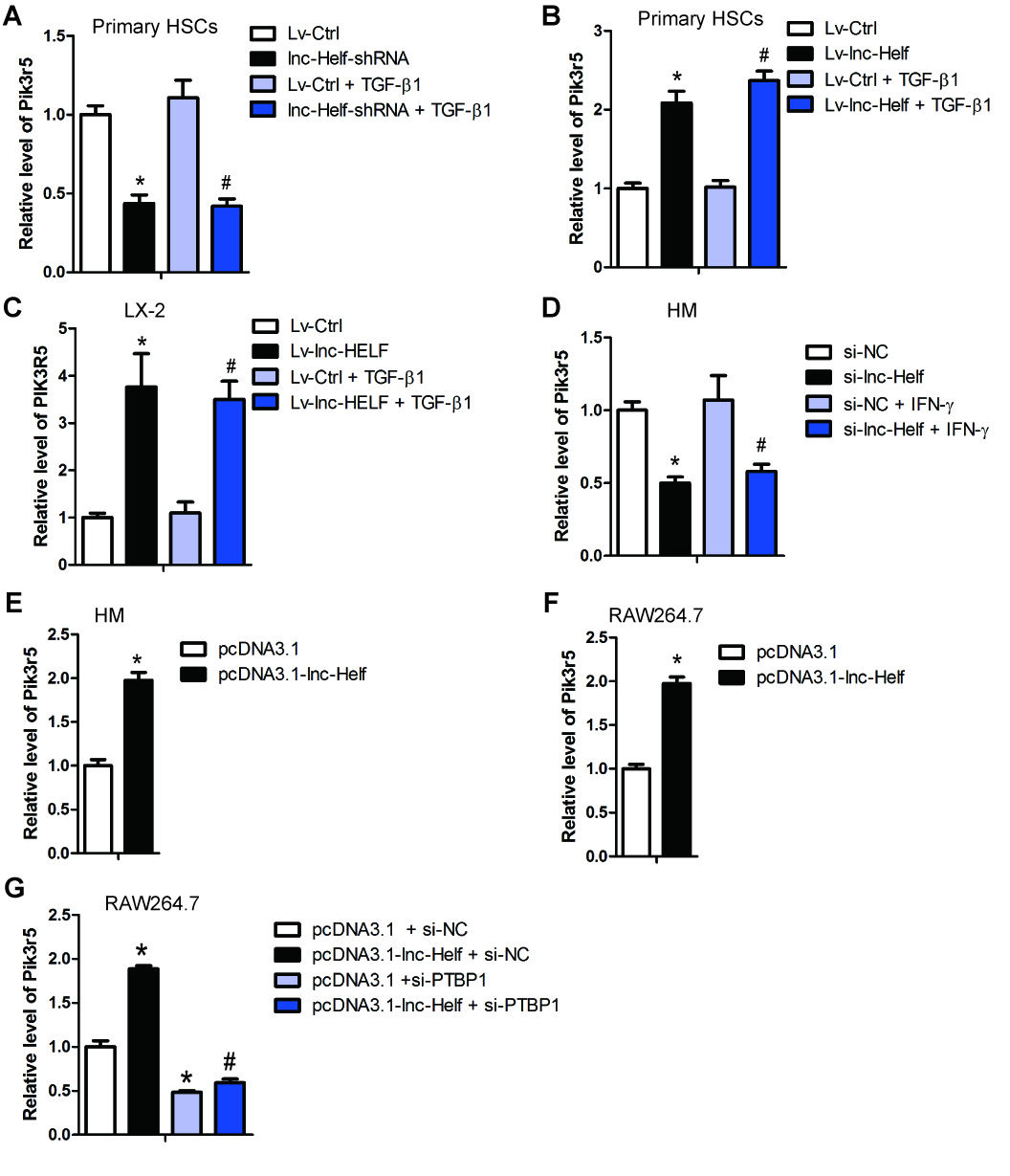
**

**Supplementary Figure 11, related to Figure 8.** (A, B) Primary HSCs at day 2 were infected with lenti-Ctrl or lnc-Helf-shRNA or lenti-lnc-Helf for 48 hours, following by the treatment of TGF-β1 for 24 hours. qRT-PCR was used to detect the RNA level of *Pik3r5*. (C) LX-2 cells were infected with lenti-Ctrl or lenti-lnc-HELF for 48 hours, following by the treatment of TGF-β1 for 24 hours. qRT-PCR was used to assess the expression of *PIK3R5*. (D) Mouse primary HMs were transfected with lnc-Helf siRNA for 24 hours following treatment of 20ng/ml IFN-γ for 24 hours. qRT-PCR was used to assess the expression of *Pik3r5*. (E, F) Mouse primary HMs and RAW264.7 cells were transfected with pcDNA3.1 or pcDNA3.1-lnc-Helf for 48 hours, qRT-PCR was used to assess the expression of *Pik3r5*. (G) qRT-PCR was used to assess the expression of *Pik3r5* in lnc-Helf-over-expressed RAW264.7 cells simultaneously transfected with siPTBP1. Data are presented as mean ± SEM. ^*/#^*p* < 0.05. **p*<0.05 for vs Lv-Ctrl or si-NC or pcDNA3.1or pcDNA3.1 + si-NC. ^#^*p*<0.05 for vs Lv-Ctrl + TGF-β1/IFN-γ or pcDNA3.1-lnc-Helf + si-NC, one-way ANOVA (A-D, and G) and Unpaired Student’s t test (E, and F).


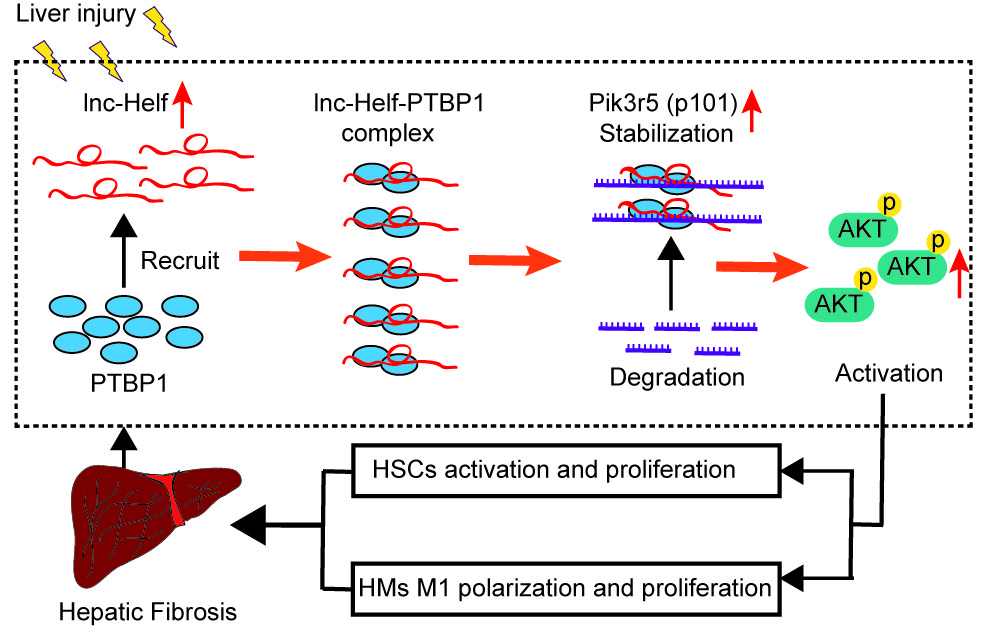


**Supplementary Figure 12.** Schematic diagram shows the function and mechanism of lnc-Helf in the progression of hepatic inflammation and fibrosis. Upon liver injury, increased lnc-Helf binds with PTBP1 to promote its interaction with PIK3R5 mRNA, resulting in increased stability and activating the AKT pathway, thus promotes HSCs and HMs activation and proliferation, which augments hepatic inflammation and fibrosis.

**Supplementary Tables**

**Supplementary Table1. Clinical characteristics of patients**

| METAVIR score | Normal  F0 | Fibrosis | | | |
| --- | --- | --- | --- | --- | --- |
|  |  | **F1** | **F2** | **F3** | **F4** |
| Cases(n) | 6 | 7 | 9 | 6 | 6 |
| Age (years)* | 57.7 ±15.7 | 52.4 ±9.9 | | | |
| Male sex (*n* (%)) | 4 (66.7) | 14 (50) | | | |
| ALT (U/L)* | 22.0 ± 12.3 | 33.87 ± 25.8 | | | |
| AST (U/L)* | 26.5 ± 12.6 | 51.3 ± 64.5 | | | |
| GGT (U/L)* | 47.3 ± 25.0 | 94.1 ± 72.6 | | | |
| Etiology (*n* (%)) |  |  | | | |
| Biliary Obstruction | 0 (0) | 2 (7.1) | | | |
| HBV | 0 (0) | 25 (89.3) | | | |
| HCV | 0 (0) | 1 (3.6) | | | |

*Mean ± SD.

ALT, alanine aminotransferase; AST, aspartate aminotransferase; GGT, γ-glutamyl transpeptidase; HBV, hepatitis B virus; HCV, hepatitis C virus.

**Supplementary Table 2. The results of mass spectrometry from proteins pulled down by the sense and antisense of lnc-Helf**

| Accession | Gene Name | Description | Coverage (%) | #Unique peptides | MW [kDa] |
| --- | --- | --- | --- | --- | --- |
| E9Q5B6 | Hnrnpd | Heterogeneous nuclear ribonucleoprotein D0 (Fragment) OS=Mus musculus GN=Hnrnpd PE=1 SV=1 - [E9Q5B6_MOUSE] | 22.43 | 2 | 12.1 |
| Q8CB58 | **Ptbp1** | MCG13402, isoform CRA_d OS=Mus musculus GN=Ptbp1 PE=1 SV=1 - [Q8CB58_MOUSE] | 17.16 | 2 | 52.6 |
| A2AC16 | Dcxr | Dicarbonyl L-xylulose reductase, isoform CRA_a OS=Mus musculus GN=Dcxr PE=4 SV=1 - [A2AC16_MOUSE] | 16.10 | 3 | 25.0 |
| P62204 | Calm1 | Calmodulin OS=Mus musculus GN=Calm1 PE=1 SV=2 - [CALM_MOUSE] | 10.74 | 1 | 16.8 |
| P62889 | Rpl30 | 60S ribosomal protein L30 OS=Mus musculus GN=Rpl30 PE=2 SV=2 - [RL30_MOUSE] | 10.43 | 1 | 12.8 |
| P10922 | H1f0 | Histone H1.0 OS=Mus musculus GN=H1f0 PE=2 SV=4 - [H10_MOUSE] | 9.79 | 2 | 20.8 |
| P53994 | Rab2a | Ras-related protein Rab-2A OS=Mus musculus GN=Rab2a PE=1 SV=1 - [RAB2A_MOUSE] | 9.43 | 2 | 23.5 |
| Q91X91 | Qprt | Nicotinate-nucleotide pyrophosphorylase [carboxylating] OS=Mus musculus GN=Qprt PE=2 SV=1 - [NADC_MOUSE] | 9.36 | 2 | 31.5 |
| Q923D2 | Blvrb | Flavin reductase (NADPH) OS=Mus musculus GN=Blvrb PE=2 SV=3 - [BLVRB_MOUSE] | 9.22 | 2 | 22.2 |
| Q9D0S9 | Hint2 | Histidine triad nucleotide-binding protein 2, mitochondrial OS=Mus musculus GN=Hint2 PE=1 SV=1 - [HINT2_MOUSE] | 9.20 | 1 | 17.3 |
| Q60605 | Myl6 | Myosin light polypeptide 6 OS=Mus musculus GN=Myl6 PE=1 SV=3 - [MYL6_MOUSE] | 8.61 | 1 | 16.9 |
| Q9DD20 | Mettl7b | Methyltransferase-like protein 7B OS=Mus musculus GN=Mettl7b PE=2 SV=2 - [MET7B_MOUSE] | 8.20 | 2 | 28.0 |
| E9Q6L3 | Auh | Methylglutaconyl-CoA hydratase, mitochondrial OS=Mus musculus GN=Auh PE=1 SV=1 - [E9Q6L3_MOUSE] | 7.83 | 1 | 12.3 |
| Q9CRB3 | Urah | 5-hydroxyisourate hydrolase OS=Mus musculus GN=Urah PE=1 SV=1 - [HIUH_MOUSE] | 7.63 | 1 | 13.6 |
| Q60759 | Gcdh | Glutaryl-CoA dehydrogenase, mitochondrial OS=Mus musculus GN=Gcdh PE=1 SV=2 - [GCDH_MOUSE] | 7.53 | 3 | 48.6 |
| A8Y5N4 | Hsd17b13 | 17-beta-hydroxysteroid dehydrogenase 13 OS=Mus musculus GN=Hsd17b13 PE=1 SV=1 - [A8Y5N4_MOUSE] | 7.20 | 2 | 29.4 |
| P60335 | Pcbp1 | Poly(rC)-binding protein 1 OS=Mus musculus GN=Pcbp1 PE=1 SV=1 - [PCBP1_MOUSE] | 7.02 | 2 | 37.5 |
| P16331 | Pah | Phenylalanine-4-hydroxylase OS=Mus musculus GN=Pah PE=1 SV=4 - [PH4H_MOUSE] | 5.96 | 3 | 51.9 |
| Q8VBW8 | Ttc36 | Tetratricopeptide repeat protein 36 OS=Mus musculus GN=Ttc36 PE=2 SV=1 - [TTC36_MOUSE] | 5.91 | 1 | 20.1 |
| F8WIT2 | Anxa6 | Annexin OS=Mus musculus GN=Anxa6 PE=1 SV=1 - [F8WIT2_MOUSE] | 5.85 | 4 | 75.2 |
| E9PXC3 | Cyp2c69 | Protein Cyp2c69 OS=Mus musculus GN=Cyp2c69 PE=3 SV=1 - [E9PXC3_MOUSE] | 5.50 | 2 | 56.1 |
| Q8BTY1 | Ccbl1 | Kynurenine--oxoglutarate transaminase 1 OS=Mus musculus GN=Ccbl1 PE=1 SV=1 - [KAT1_MOUSE] | 4.95 | 2 | 47.5 |
| D3YTT4 | Acad8 | Isobutyryl-CoA dehydrogenase, mitochondrial OS=Mus musculus GN=Acad8 PE=1 SV=1 - [D3YTT4_MOUSE] | 4.84 | 2 | 45.0 |
| Q9JI75 | Nqo2 | Ribosyldihydronicotinamide dehydrogenase [quinone] OS=Mus musculus GN=Nqo2 PE=2 SV=3 - [NQO2_MOUSE] | 4.76 | 1 | 26.2 |
| B1AWE0 | Clta | Clathrin light chain A OS=Mus musculus GN=Clta PE=1 SV=1 - [B1AWE0_MOUSE] | 4.17 | 1 | 23.5 |
| Q9D6R2-2 | Idh3a | Isoform 2 of Isocitrate dehydrogenase [NAD] subunit alpha, mitochondrial OS=Mus musculus GN=Idh3a - [IDH3A_MOUSE] | 4.17 | 1 | 31.4 |
| F6SAC3 | Gm1840 | Glucose-6-phosphate isomerase OS=Mus musculus GN=Gm1840 PE=3 SV=1 - [F6SAC3_MOUSE] | 4.03 | 2 | 53.2 |
| Q9EQF5 | Dpys | Dihydropyrimidinase OS=Mus musculus GN=Dpys PE=1 SV=2 - [DPYS_MOUSE] | 3.66 | 1 | 56.7 |
| Q9DC50 | Crot | Peroxisomal carnitine O-octanoyltransferase OS=Mus musculus GN=Crot PE=1 SV=1 - [OCTC_MOUSE] | 3.43 | 2 | 70.2 |
| Q76MZ3 | Ppp2r1a | Serine/threonine-protein phosphatase 2A 65 kDa regulatory subunit A alpha isoform OS=Mus musculus GN=Ppp2r1a PE=1 SV=3 - [2AAA_MOUSE] | 3.40 | 2 | 65.3 |
| E9Q1Y9 | 5430421N21Rik | Protein 5430421N21Rik OS=Mus musculus GN=5430421N21Rik PE=3 SV=1 - [E9Q1Y9_MOUSE] | 2.92 | 1 | 52.8 |
| P47791-2 | Gsr | Isoform Cytoplasmic of Glutathione reductase, mitochondrial OS=Mus musculus GN=Gsr - [GSHR_MOUSE] | 2.53 | 1 | 51.0 |
| Q02053 | Uba1 | Ubiquitin-like modifier-activating enzyme 1 OS=Mus musculus GN=Uba1 PE=1 SV=1 - [UBA1_MOUSE] | 2.46 | 2 | 117.7 |
| Q9DBL1 | Acadsb | Short/branched chain specific acyl-CoA dehydrogenase, mitochondrial OS=Mus musculus GN=Acadsb PE=1 SV=1 - [ACDSB_MOUSE] | 2.31 | 1 | 47.8 |
| Q8C165 | Pm20d1 | Probable carboxypeptidase PM20D1 OS=Mus musculus GN=Pm20d1 PE=2 SV=1 - [P20D1_MOUSE] | 1.79 | 1 | 55.6 |
| Q921I1 | Trfe | Serotransferrin OS=Mus musculus GN=Tf PE=1 SV=1 - [TRFE_MOUSE] | 1.72 | 1 | 76.7 |
| Q68FD5 | Cltc | Clathrin heavy chain 1 OS=Mus musculus GN=Cltc PE=1 SV=3 - [CLH1_MOUSE] | 1.67 | 2 | 191.4 |
| Q91W43 | Gldc | Glycine dehydrogenase (decarboxylating), mitochondrial OS=Mus musculus GN=Gldc PE=1 SV=1 - [GCSP_MOUSE] | 1.07 | 1 | 113.2 |
| Q9QUQ5-2 | Trpc4 | Isoform Beta of Short transient receptor potential channel 4 OS=Mus musculus GN=Trpc4 - [TRPC4_MOUSE] | 1.01 | 1 | 102.1 |
| O54991 | Cntnap1 | Contactin-associated protein 1 OS=Mus musculus GN=Cntnap1 PE=2 SV=2 - [CNTP1_MOUSE] | 0.58 | 1 | 156.2 |

**Supplementary Table 3. Cloning primers for lnc-Helf**

| Name | Sequence 5’-3’ |
| --- | --- |
| Mouse lnc-Helf BamH1 F | cgcggatccAGGGGGAACTCTTCTGCCAA |
| Mouse lnc-Helf BamH1 R | cgcggatccATACACAATAAAGAGGTTCACAGCG |
| Human lnc-HELF BamH1 F | cgcggatccACAACCAAGGGAGAAAACTA |
| Human lnc-HELF BamH1 R | cgcggatccTAACAAAGAGTTTCACAGCAG |
| Mouse lnc-Helf BamH1 F | cgcggatccAGGGGGAACTCTTCTGCCAA |
| Mouse lnc-Helf Xho1 R | ccgctcgagATACACAATAAAGAGGTTCACAGCG |
| Mouse sh-lnc-Helf F1 | GATCCCCGACCTTTCTTACCTCAGAATTCAAGAGATTCTGAGGTAAGAAAGGTCTTTTTA |
| Mouse sh-lnc-Helf R1 | AGCTTAAAAAGACCTTTCTTACCTCAGAATCTCTTGAATTCTGAGGTAAGAAAGGTCGGG |
| Mouse sh-lnc-Helf F2 | GATCCCCGCTAACCAGGAAATGAACTTTCAAGAGAAGTTCATTTCCTGGTTAGCTTTTTA |
| Mouse sh-lnc-Helf R2 | AGCTTAAAAAGCTAACCAGGAAATGAACTTCTCTTGAAAGTTCATTTCCTGGTTAGCGGG |
| Mouse sh-lnc-Helf F3 | GATCCCCCCGCTGTGAACCTCTTTATTTCAAGAGAATAAAGAGGTTCACAGCGGTTTTTA |
| Mouse sh-lnc-Helf R3 | AGCTTAAAAACCGCTGTGAACCTCTTTATTCTCTTGAAATAAAGAGGTTCACAGCGGGGG |
| Human sh-lnc-HELF F1 | GATCCCCGAACAGGTTGTGTTGTTATTTCAAGAGAATAACAACACAACCTGTTCTTTTTA |
| Human sh-lnc-HELF R1 | AGCTTAAAAAGAACAGGTTGTGTTGTTATTCTCTTGAAATAACAACACAACCTGTTCGGG |
| Human sh-lnc-HELF F2 | GATCCCCGGGATTCTTCTCAAGATATTTCAAGAGAATATCTTGAGAAGAATCCCTTTTTA |
| Human sh-lnc-HELF R2 | AGCTTAAAAAGGGATTCTTCTCAAGATATTCTCTTGAAATATCTTGAGAAGAATCCCGGG |
| Human sh-lnc-HELF F3 | GATCCCCGCTGTGAAACTCTTTGTTATTCAAGAGATAACAAAGAGTTTCACAGCTTTTTA |
| Human sh-lnc-HELF R3 | AGCTTAAAAAGCTGTGAAACTCTTTGTTATCTCTTGAATAACAAAGAGTTTCACAGCGGG |
| AAV8- sh-lnc-Helf F1 | GATCCCCGACCTTTCTTACCTCAGAATTCAAGAGATTCTGAGGTAAGAAAGGTCTTTTTG |
| AAV8- sh-lnc-Helf R1 | AATTCAAAAAGACCTTTCTTACCTCAGAATCTCTTGAATTCTGAGGTAAGAAAGGTCGGG |
| AAV8- sh-lnc-Helf F2 | GATCCCCGCTAACCAGGAAATGAACTTTCAAGAGAAGTTCATTTCCTGGTTAGCTTTTTG |
| AAV8- sh-lnc-Helf R2 | AATTCAAAAAGCTAACCAGGAAATGAACTTCTCTTGAAAGTTCATTTCCTGGTTAGCGGG |
| AAV8- sh-lnc-Helf F3 | GATCCCCGCCGCTGTGAACCTCTTTATTTCAAGAGAATAAAGAGGTTCACAGCGGTTTTTG |
| AAV8- sh-lnc-Helf R3 | AATTCAAAAACCGCTGTGAACCTCTTTATTCTCTTGAAATAAAGAGGTTCACAGCGGCGGG |
| Negative control F | GATCCCCGTTCTCCGAACGTGTCACGTTCAAGAGACGTGACACGTTCGGAGAACTTTTTA |
| Negative control R | AGCTTAAAAAGTTCTCCGAACGTGTCACGTCTCTTGAACGTGACACGTTCGGAGAACGGG |

**Supplementary Table 4. siRNA sequences**

| Name | Forward 5' - 3' |
| --- | --- |
| si-lnc-Helf-1 | GACCUUUCUUACCUCAGAATT |
| si-lnc-Helf-2 | GCTAACCAGGAAATGAACTTT |
| si-lnc-Helf-3 | CCGCUGUGAACCUCUUUAUTT |
| si-PTBP1 | GGGUGAAGAUCCUGUUCAATT |
| negative control | UUCUCCGAACGUGUCACGUTT |

**Supplementary Table 5. qRT-PCR primers**

| Gene symbol | Forward 5' - 3' | Reverse 5 '- 3' |
| --- | --- | --- |
| Mouse Gapdh | GGCATGGACTGTGGTCATGAG | TGCACCACCAACTGCTTAGC |
| Mouse lnc-Helf | GTTGTGGTGATTGGAGCAG | CAGCGTGACCTATTCTGAGG |
| Mouse Acta2 | TCGGATACTTCAGCGTCAGGA | GTCCCAGACATCAGGGAGTAA |
| Mouse Col1α1 | ATCGGTCATGCTCTCTCCAAACA | ACTGCAACATGGAGACAGGTCAGA |
| Mouse Col1α2 | TGGCAGAGCTGGTGTAATGG | TAGGACCTCGGATTCCAGCA |
| Mouse Timp1 | TCCGTCCACAAACAGTGAGTGTCA | GGTGTGCACAGTGTTTCCCTGTTT |
| Mouse Mmp2 | GTGTTCTTCGCAGGGAATGAG | GATGCTTCCAAACTTCACGCT |
| Mouse Tgf-β1 | GGACTCTCCACCTGCAAGAC | CATAGATGGCGTTGTTGCGG |
| Mouse Pcna | TTTGAGGCACGCCTGATCC | GGAGACGTGAGACGAGTCCAT |
| Mouse Ki67 | CATCCATCAGCCGGAGTCA | TGTTTCGCAACTTTCGTTTGTG |
| Mouse Bcl-2 | GCTGGGATGCCTTTGTGGAACT | CAGAGACAGCCAGGAGAAATCAAAC |
| Mouse CyclinD1 | TCAAGTGTGACCCGGACTG | ATGTCCACATCTCGCACGTC |
| Mouse CyclinE1 | GCTGCTAAGGAGGGTGCTAC | AGCAACCTACAACACCCGAG |
| Mouse Il-1β | GTCGCTCAGGGTCACAAGAA | GTG-CTGCCTAATGTCCCCTT |
| Mouse Ly6c | GCAGTGCTACGAGTGCTATGG | ACTGACGGGTCTTTAGTTTCCTT |
| Mouse Il-6 | AGTTGCCTTCTTGGGACTGA | TCCACGATTTCCCAGAGAAC |
| Mouse Tnf-α | CATCTTCTCAAAATTCGAGTGACAA | TGGGAGTAGACAAGGTACAACCC |
| Mouse Mcp1 | GTTAACGCCCCACTCACCTG | GGGCCGGGGTATGTAACTCA |
| Mouse Neat1 | GGGAAGGGTGTGGTCAGAAG | GGCAGGTTGGCTCCTACAAT |
| Mouse Pik3r5 | CTCACCCCAACTGCTGAGAGTC | CCGAGGCTCAGTCCATGCAA |
| Mouse Ptbp1 | GCTGGGAATTCTGTCCTTTTG | ACGCCGAAGAGAATAAAGAGG |
| Homo GAPDH | ACCCAGAAGACTGTGGATGG | TTCAGCTCAGGGATGACCTT |
| Homo lnc-HELF | TGGGTCTTGAACAGGTTGTG | AGAGATGCCAGCCTATGACT |
| Homo PIK3R5 | AAGTCCTGTGCCAGCAGTC | TCTGTCGTCTTCTCCCCAGT |
| Homo PTBP1 | AGCGCGTGAAGATCCTGTTC | CAGGGGTGAGTTGCCGTAG |
| Homo PCNA | TCCATCCTCAAGAAGGTGTT | GGTAGGTGTCGAAGCCC |
| Homo KI67 | ACCCTGATGAGAGTGAGGGA | GGGGCTTCTCCCCTTTTGAG |
| Homo CYCLIN D1 | GTGGCCTCTAAGATGAAGGAGA | GGAAGTGTTCAATGAAATCGTG |
| Homo ACTA2 | GCCATGTTCTATCGGGTACTTC | CAGGGCTGTTTTCCCATCCAT |
| Homo COL1α1 | AACCAAGGCTGCAACCTGGA | GGCTGAGTAGGGTACACGCAGG |
| Homo COL1α2 | GCAGGAGGTTTCGGCTAAGT | GCAACAAAGTCCGCGTATCC |
| Homo TIMP1 | GGGGCTTCACCAAGACCTAC | GGAAGCCCTTTTCAGAGCCT |
| Homo TGF-β1 | TGTTGGACAGCTGCTCCACCT | GGCAGTGGTTGAGCCGTGGA |

**Supplementary Table 6. RACE primers for lnc-Helf**

| gene specific primer | Sequence 5' - 3' |
| --- | --- |
| 3' OUTER PRIMER | GATTACGCCAAGCTTTGGTGATTGGAGCAGGGGTCAGTGGCCT |
| 5' OUTER PRIMER | GATTACGCCAAGCTTGGGGTCCATTCTCTGAAGAAGCACTTTGC |
| 5' INNER PRIMER | GATTACGCCAAGCTTACACGGGCTGTTTTGCTTTAGCTGGA |
